# Identification of antimalarial targets of chloroquine by a combined deconvolution strategy of ABPP and MS-CETSA

**DOI:** 10.1186/s40779-022-00390-3

**Published:** 2022-06-14

**Authors:** Peng Gao, Yan-Qing Liu, Wei Xiao, Fei Xia, Jia-Yun Chen, Li-Wei Gu, Fan Yang, Liu-Hai Zheng, Jun-Zhe Zhang, Qian Zhang, Zhi-Jie Li, Yu-Qing Meng, Yong-Ping Zhu, Huan Tang, Qiao-Li Shi, Qiu-Yan Guo, Ying Zhang, Cheng-Chao Xu, Ling-Yun Dai, Ji-Gang Wang

**Affiliations:** 1 Artemisinin Research Center, Institute of Chinese Materia Medica, China Academy of Chinese Medical Sciences, Beijing, 100700 China; 2 Department of Traditional Chinese Medicine, Southern Medical University, Guangzhou, 510515 China; 3 Department of Geriatrics, the Second Clinical Medical College of Jinan University, the First Affiliated Hospital of Southern University of Science and Technology, Shenzhen People’s Hospital, Shenzhen, 518020 Guangdong China; 4 Institute of Molecular and Cell Biology, Agency for Science, Technology and Research (A*STAR), Singapore, 138673 Singapore; 5 Department of Biological Sciences, National University of Singapore, Singapore, 117543 Singapore

**Keywords:** Chloroquine, Antimalaria, Activity-based protein profiling (ABPP), Cellular thermal shift assay (CETSA), Quantitative proteomics

## Abstract

**Background:**

Malaria is a devastating infectious disease that disproportionally threatens hundreds of millions of people in developing countries. In the history of anti-malaria campaign, chloroquine (CQ) has played an indispensable role, however, its mechanism of action (MoA) is not fully understood.

**Methods:**

We used the principle of photo-affinity labeling and click chemistry-based functionalization in the design of a CQ probe and developed a combined deconvolution strategy of activity-based protein profiling (ABPP) and mass spectrometry-coupled cellular thermal shift assay (MS-CETSA) that identified the protein targets of CQ in an unbiased manner in this study. The interactions between CQ and these identified potential protein hits were confirmed by biophysical and enzymatic assays.

**Results:**

We developed a novel clickable, photo-affinity chloroquine analog probe (CQP) which retains the antimalarial activity in the nanomole range, and identified a total of 40 proteins that specifically interacted and photo-crosslinked with CQP which was inhibited in the presence of excess CQ. Using MS-CETSA, we identified 83 candidate interacting proteins out of a total of 3375 measured parasite proteins. At the same time, we identified 8 proteins as the most potential hits which were commonly identified by both methods.

**Conclusions:**

We found that CQ could disrupt glycolysis and energy metabolism of malarial parasites through direct binding with some of the key enzymes, a new mechanism that is different from its well-known inhibitory effect of hemozoin formation. This is the first report of identifying CQ antimalarial targets by a parallel usage of labeled (ABPP) and label-free (MS-CETSA) methods.

**Supplementary Information:**

The online version contains supplementary material available at 10.1186/s40779-022-00390-3.

## Background

Malaria is an ancient lethal infectious disease that is still widely distributed in nearly 90 countries, mainly in developing countries, around the world [1]. According to the World Malaria Report 2021, there were about 241 million malaria infection cases in 2020, claiming 627,000 lives [2]. In the history of antimalarial drug development, the discovery of chloroquine (CQ) represented a major breakthrough and CQ has played an essential role in the past anti-malaria campaign [3]. As a long-acting, cost-effective, and well-tolerated drug, CQ is still prescribed to prevent and treat the infection of *Plasmodium vivax* (*P. vivax*), *Plasmodium ovale*, *Plasmodium malariae*, and *Plasmodium knowlesi*, but not *Plasmodium falciparum* (*P. falciparum*) in which CQ resistance unfortunately becomes a widespread phenomenon [4]. Of note, since the first report of CQ-resistant *P. vivax* in 1989 [5], although slowly, many regions of the world have seen the increasing emergence of CQ resistance by *P. vivax* [6]. Despite the urgency, our understanding of the cellular and molecular determinants for this resistance is still limited [7].

The nutrients needed for the rapid growth and reproduction of malaria parasites mainly come from the degradation of erythrocyte hemoglobin proteins in the digestive vacuole (DV), which can also create space for the growth of parasites and prevent the host red blood cells (RBCs) from dissolving prematurely [8]. However, the digestion of hemoglobin is accompanied by the production of toxic soluble molecule heme, which is also an activator of artemisinin drugs [9, 10]. The existence of high concentration of heme and the acidic environment in DV are conducive to the Fenton reactions to produce a large number of oxygen free radicals, resulting in protein denaturation, DNA damage, cell membrane destruction, and the death of parasites [11, 12]. Malarial parasites rely on the formation of inert crystalline hemozoin to sequester the toxic heme [13]. CQ is a diprotic weak-basic drug (pKa1 = 8.1, pKa2 = 10.2) that could exist in protonated and unprotonated forms [14]. Once entering the DV of the parasite, CQ becomes protonated and charged, resulting in its trapping and accumulation inside this acidic organelle [14]. Interestingly, CQ is able to not only disrupt this heme detoxification process by inhibiting the growth of hemozoin crystal, but also puncture the DV membrane by forming a complex with the free heme [15].

Moreover, CQ as well its derivative hydroxychloroquine (HCQ) are also used to treat autoimmune disorders, including rheumatoid arthritis and systematic lupus erythematosus [16]. The mechanism of action (MoA) of CQ and MoA of HCQ in these settings are starting to be revealed, probably through interference with lysosomal activity and autophagy [17].

Despite decades of extensive research, the MoA responsible for CQ’s antimalarial effect is not fully understood. To this end, a system-wide method is needed. Here, we systematically identified the protein targets of CQ by a combined deployment of activity-based protein profiling (ABPP) technology [10, 18–20] and mass spectrometry-coupled cell thermal shift analysis (MS-CETSA) [21–23], for a more complete understanding of its antimalarial effect at the proteome level.

## Methods

### Synthesis of chloroquine analog probe (CQP)

Synthetic steps and intermediate products of CQP are described in Additional file 1, as are the NMR and MS Spectra.

### Parasite culture

The *P. falciparum* 3D7 strain was obtained from Artemisinin Research Center of China, Academy of Chinese Medical Sciences. Parasites were cultured in malaria culture media (MCM) containing 10.4 g/L RPMI 1640 (Gibco, CA, USA), 0.5% albumin (MP, Santa Ana, CA, USA), 0.2 g/L gentamycin (Sangon, Shanghai, China), 25 µg/ml hypoxanthine (Sigma, St Louis, Missouri, USA), 0.3 g/L L-glutamine (Sigma, St Louis, Missouri, USA), 25 mmol/L HEPES (Avantor, Radnor, PA, USA), 2.5 g/L NaHCO_3_ (Sigma, St Louis, Missouri, USA) and supplement of 2% healthy human erythrocytes provided by Beijing Red Cross Blood Center. Parasites were maintained at 37 °C with 5% CO_2_, 5% O_2_, and 90% N_2_. Giemsa-stained thin blood smears were performed to evaluate the parasite growth status and parasitemia [24].

### Antimalarial activity assay

The antimalarial activities of compounds were measured using a fluorescent SYBR Green I based assay as described previously with slight modifications [25]. Briefly, parasites were synchronized using 5% sorbitol twice, and then the highly synchronized ring-stage parasites were prepared at 2% hematocrit and 0.5% parasitemia. Parasite cultures were treated with threefold serially diluted concentrations of compounds starting from 30 µmol/L in 96-well microplates for 72 h at 37 °C with 5% CO_2_, 5% O_2_, and 90% N_2_. Subsequently, 100 µl lysis buffer (30 mmol/L Tris pH 7.5, 10 mmol/L EDTA, 0.01% saponin, 0.08% Triton X-100) containing 2 × SYBR Green I (Thermo, Waltham, Mass, USA) was added in each well for 1.5 h in dark at 37 °C. Then, the fluorescence was measured using EnVision 2105 Multimode Plate Reader (PerkinElmer, Waltham, Mass, USA) with λ_ex_ = 485 nm and λ_em_ = 535 nm. Uninfected erythrocytes and drug-free infected erythrocytes (0.5% parasitemia) with the same hematocrit were served as blank control and negative control, respectively. Three biological replicates and technical replicates were performed for each drug dose. The half-maximal inhibitory concentration (IC_50_) was calculated and dose–response curve fitting of the % inhibition vs. log (dose) was performed using the GraphPad Prism 8.

### Fluorescence labeling assay of *P. falciparum*

The fluorescence labelling assay was carried out following the published method with slight modifications [10]. For in situ fluorescence labelling assay, parasites were cultured in 6-well plate with 4 ml complete medium, and adjusted to 5% parasitemia with 2% hematocrit. Following incubation with increasing concentrations (0.2–20 µmol/L) of CQP dissolved in dimethyl sulfoxide (DMSO, final concentration was 0.1%) for 4 h, the culture plates were subject to UV irradiation (λ = 365 nm) for 10 min on ice.

To release parasites from the infected RBCs (iRBCs), the cultures were pelleted down and incubated with 10 volumes of cold 0.05% saponin containing 1 × Halt™ protease inhibitor (PI) cocktail (Thermo, Waltham, Mass, USA) for 10 min on ice. Parasites were pelleted at 4000 rpm for 10 min at 4 °C before being washed 3 times with 10 volumes of pre-chilled PBS containing 1 × PI. The pellet was resuspended with lysis buffer containing 1 × PI, 50 mmol/L HEPES, 5 mmol/L β-glycerophosphate, 0.1 mmol/L activated Na_3_VO_4_, 20 mmol/L MgCl_2_, and 1 mmol/L Tris (2-carboxyethyl) phosphine (TCEP). The resulting pellet was subject to 3 cycles of flash-freeze–thawing in liquid nitrogen and room temperature (RT) water, followed by brief sonication on ice water. The soluble protein fraction in the supernatant was collected and the protein concentrations were determined using Bicinchoninic Acid Protein Assay Kit (Thermo Scientific, Waltham, Mass, USA).

The lysate samples were then labeled with a tetramethyl-6-carboxyrhodamine azide (TAMRA-azide) fluorescent tag (Additional file 2: Fig. S1a) through the copper catalyzed azide alkyne cycloaddition (CuAAC) click chemistry reaction using Tris[(1-benzyl-1H-1,2,3triazol-4-yl) methyl] amin (TBTA) (100 µmol/L in DMSO), TCEP (1 mmol/L in water), CuSO_4_ (1 mmol/L in water) and TAMRA-azide (50 µmol/L in DMSO) purchased from Click chemistry tools (Arizona, AZ, USA). Pre-chilled ice acetone was added to precipitate the proteins. Then, 30 µl of 1 × SDS-PAGE loading buffer (Beyotime, Beijing, China) was added to dissolve the proteins by sonication and heating for 10 min at 95 °C. Furthermore, 10 µl per sample was loaded onto 10% acrylamide gel, which was made using Acryl/Bis 40% Solution (29:1), 4 × Tris–HCl/SDS (pH 8.8), 4 × Tris–HCl/SDS (pH 6.8), 10% ammonium persulfate and 1,2-Bis (dimethylamino) ethane (Solarbio, Beijing, China). The fluorescence scanning (λ_ex_ = 550 nm and λ_em_ = 570 nm) and image analysis were performed using Sapphire Biomolecular Imager (Azure Biosystems, San Diego, California, USA), following stained with Coomassie brilliant blue for loading control.

For in vitro fluorescence labelling assay of parasite lysates, parasites (unsynchronized) were cultured to approximately 10% parasitemia with 2% hematocrit. The soluble parasite lysates were prepared as described above. Equal amount of protein lysates was incubated with increasing concentrations (10–2000 nmol/L) of CQP for 4 h at RT, and equal volume DMSO was served as blank control with a final concentration of 0.1%. For competitive labeling assay, the protein lysate was pre-treated with up to 50-fold excess of CQ for 1 h, followed by 2 µmol/L CQP for 4 h. Samples were then irradiated with UV light (λ = 365 nm) for 10 min on ice, and the unirradiated samples with the same treatment were used as parallel negative control. Finally, the probe labelled proteins were subject to fluorescence labeling, SDS-PAGE separation and fluorescence scanning.

### Target identification through parasite labeling and ABPP

The ABPP experiment was carried out as described previously [10]. The extraction of parasite lysate proteins was carried out as described above. Parasite lysate proteins (500 µg) were incubated with CQP (2 µmol/L) for 4 h at RT, with equal volume of DMSO as negative control. A competition assay was performed by pre-treating the lysate with 200 µmol/L CQ for 1 h followed by 2 µmol/L CQP for 4 h. The samples were irradiated with UV light (λ = 365 nm) for 10 min on ice. The click chemistry was carried out to conjugate biotin tag (Additional file 2: Fig. S1b) to proteins using TBTA (100 µmol/L in DMSO), TCEP (1 mmol/L in water), CuSO_4_ (1 mmol/L in water) and biotin-azide (50 µmol/L). Then samples were precipitated with acetone again and redissolved in 0.1% SDS in PBS, followed by incubation with 60 µl NeutrAvidin beads (Thermo Scientific, Waltham, Mass, USA) at RT for 4 h with gentle rotation.

The beads were then washed with 1% SDS, 6 mol/L urea and 1 × PBS three times, respectively; and incubated with denaturation and reduction buffer containing 500 µl of 6 mol/L urea and 25 µl of 100 mmol/L dithiothreitol for 30 min at 37 °C, followed by alkylation with 25 µl of 400 mmol/L iodoacetamide in dark for 30 min at RT. The beads were then incubated with 150 µl of 2 mol/L urea with 1 mmol/L CaCl_2_ and 3 µg trypsin at 37 °C overnight. After the digestion, the supernatants containing peptides were desalted using C18 column (Waters, Milford, Mass, USA). Samples were labeled with TMT10plex Labeling Reagents (Thermo Scientific, Waltham, Mass, USA) for 4 h at RT, and analysed by liquid chromatography-tandem mass spectrometry (LC–MS/MS).

### LC–MS/MS measurement and data analysis

LC–MS/MS analyses were performed on an UltiMate 3000 RSLC nano-LC system coupled with an Orbitrap Fusion Lumos Mass Spectrometer (Thermo Fisher Scientific, Waltham, Mass, USA). Dried peptide sample fractions were reconstituted in 0.1% formic acid and 1% acetonitrile, and separated on an Acclaim™ PepMap™100 C18 analytical column (130 Å, 2 µm, 75 µm × 250 mm). The mass spectrometer was operated in the data-dependent acquisition mode. All MS spectra detection was performed in positive-ion mode with charge states of 2–6 included, using orbitrap detector with a full scan MS spectra range of 300–1500 m/z at 60,000 resolutions. The top 20 most abundant precursors were subject to high-energy collision-induced dissociation.

The mass spectrometry raw data were analyzed by Proteome Discoverer (PD) version 2.4. The PD search parameters were set as follows: the precursor mass tolerance was set to 15 ppm and fragment mass tolerance to 0.02 Da. Carbamidomethyl/+ 57.021 Da (C) and Oxidation/+ 15.995 Da (M) were set as static modification, Deamidated/+ 0.984 Da (N, Q) was set as peptide dynamic modification and Acetyl/+ 42.011 Da was set as protein N-terminal dynamic modification. The minimum peptide length was set to 6. Peptide spectra matches were filtered with false-discovery rates of 1% (Strict) and 5% (Relaxed) on the peptide spectrum match and subsequently on the protein level.

### Targets identification using isothermal dose–response (ITDR) MS-CETSA

The ITDR MS-CETSA assay was performed to identify the targets protein as described previously with slight modification [26]. The parasite lysate used in this assay was similarly prepared as described above. Equal volume of lysate aliquots was incubated with increasing concentrations of CQ (0–300 µmol/L) for 3 min at RT, followed by dividing each sample into 3 equal portions into polymerase chain reaction tubes. The samples were heated at 37 °C, 52 °C or 61 °C, respectively for 3 min, followed by cooling at 4 °C for 3 min. The heated samples were centrifuged at 21,000 g for 30 min at 4 °C, and the supernatant was collected. Then 30 µg protein per sample was transferred into new 1.5 ml tubes, and the denaturation and reduction treatment were initiated by adding 20 mmol/L TCEP, 0.05% RapiGest and 100 mmol/L TEAB, heating for 20 min at 55 °C. The alkylation step was carried out with 55 mmol/L 2-chloroacetamide and incubated for 30 min at RT in dark. Samples were digested with 1 µg LysC for 3 h followed by 1.5 µg trypsin for 18 h at 37 °C with shaking at 200 rpm. Trifluoroacetic acid was added to hydrolyze RapiGest. Samples were centrifuged at 21,000 g for 15 min at RT and the supernatants were collected and dried with a centrifugal vacuum evaporator at 60 °C.

For the TMT-labeling, each sample of 10 µg peptide was labeled with TMT10plex reagents for 2 h in dark at RT. The labeling reactions were quenched by adding 25 µL of 1 mol/L Tris–HCl (pH 8.0). Then the labeled samples were combined and desalted using Oasis HLB column. Then peptide offline prefractionation was carried out on a Nexera LC-40D XS liquid chromatography system. Elution was carried out with buffer A of 10 mmol/L ammonium formate (pH 10.0) and buffer B of 10 mmol/L ammonium formate (pH 10.0) in 80% acetonitrile using a 120 min gradient. The fractions of 15–110 min were collected in a 96-well deep-well plate. Finally, the samples were pooled to 20 fractions, then subject to LC–MS/MS analysis.

The mineCETSA R package (https://github.com/nkdailingyun/mineCETSA) was used to analyze and visualize the CETSA data. We applied an array of stringent criteria for high confident hits selection of ITDR MS-CETSA data, including: ΔAUC > 3 × MAD (AUC: area under the curve; MAD: median absolute deviation) of heat-challenged sample normalized against nondenaturing 37 °C control, the maximal fold change (≥ 1.3) of relative protein abundance in at least one drug dose-treated sample, the dose–response curve best-fitting quality (R^2^ ≥ 0.85), the Slope of dose–response curve > 0.25 and minimal dose threshold < 5 µmol/L.

### MS-data and bioinformatics analysis

The statistical analysis and visualization were performed in *R* statistical software (version 4.1.1). The differential analysis was performed using the limma package in *R* statistical software (version 3.48.3). *P*-values were generated from the empirical Bayes test model and adjusted using Benjamini–Hochberg. The proteins with absolute fold change ≥ 1.2 and adjusted *P-*value (*P*_adj_) < 0.05 were considered to be significant differences.

Gene Ontology analysis was performed using the clusterProfiler package in R statistical software (version 3.18.1). *P*-values generated from the hypergeometric test model were adjusted using Benjamini–Hochberg. The enriched functional profiles were visualized on the basis of the count of proteins enriched and the *P*_adj_.

### Pull down and target validation by Western blotting

The parasite cultures were maintained, treated with 2 µmol/L CQP probe (with or without the pre-treatment of 100 µmol/L CQ), and irradiated with UV similarly as in situ fluorescence labelling assay described above. Parasites were released from iRBCs and lysate proteins were prepared for biotin-azide conjugation through CuAAC-based click chemistry reaction. The pull down experiment was carried out through incubation with NeutrAvidin beads at RT for 4 h with gentle rotation. The enriched proteins were eluted from beads with 1 × SDS-loading buffer by boiling at 96 °C for 10 min. The supernatants were collected and separated via SDS-PAGE gel electrophoresis. The proteins were wet transferred onto polyvinylidene fluoride membranes, blocked with 5% skim milk solution, followed by incubation with the corresponding primary and secondary antibodies (Additional file 3: Table S1). Subsequently, the Western blotting results were visualized using High-sig ECL Substrate (Tanon, Shanghai, China).

### Recombinant protein expression and purification

The genes of L-lactate dehydrogenase (*Pf*LDH, *PF3D7_1324900*), ornithine aminotransferase (*Pf*OAT, *PF3D7_0608800*), pyruvate kinase (*Pf*PyrK, *PF3D7_0626800*), phosphoglycerate kinase (*Pf*PGK, *PF3D7_0922500*) and triosephosphate isomerase (*Pf*TPI, *PF3D7_1439900*) of *P. falciparum* 3D7 were synthesized commercially with codon-optimization and were cloned into pET-28a. The plasmids were transformed into *Escherichia coli* strain BL21. The cells were cultured in Luria–Bertani medium and induced with 0.5 mmol/L isopropyl b-D-thiogalactoside at 16 °C for 16 h. Cells were collected, then resuspended in buffer A (50 mmol/L HEPES pH 7.5, 300 mmol/L NaCl, 5 mmol/L b-mercaptoethanol, 1 mmol/L PMSF) and disrupted at 12,000 p.s.i. using a JN-Mini Pro homogenizer (JNBio, Beijing, China). Whole cells and cell debris were removed by centrifugation at 16,000 rpm and the supernatant was loaded onto a Ni-nitrilotriaceate affinity column. The recombinant proteins were eluted with buffer B (50 mmol/L HEPES pH 7.5, 150 mmol/L NaCl, 150 mmol/L imidazole), and the imidazole was removed using Zeba™ Spin Desalting Columns (Thermo Scientific, Waltham, Mass, USA).

### In vitro fluorescence labeling of recombinant proteins

The experiments were essentially carried out same as described for in vitro fluorescence labelling of parasite lysate samples. The recombinant proteins (2 µg) were incubated with either increasing concentrations (0–5 µmol/L) of CQP or a fixed amount of CQP (2 µmol/L) for 4 h at RT, with equal volume of DMSO included as negative control. A competition assay was performed by pre-treating the proteins with 100 µmol/L CQ for 1 h before incubating with CQP (2 µmol/L) for another 4 h. After the incubation, the UV irradiation and the click reaction for TAMRA-azide fluorescence labeling, SDS-PAGE gel electrophoresis and fluorescence scanning were carried out as described above.

### Fluorescence staining and imaging

Parasites (unsynchronized) were cultured in 24-well plate with about 5% parasitemia, 2% hematocrit and treated with DMSO or 2 µmol/L CQP for 30 min, before being fixed with 4% paraformaldehyde and 0.004% glutaraldehyde. The fixed parasite cultures were resuspended with 200 µl of PBS and dripped onto coverslips precoated with 0.01% (w/w) polylysine, permeabilized for 10 min with 0.1% Triton-X at 37 °C, washed with PBS for 3 times, and then incorporated with TAMRA-azide fluorescent tag through CuAAC-based click chemistry reaction for 1 h at RT. The coverslips were transferred to the glass slide coated with DAPI sealing agent and imaged with Leica TCS SP8 SR confocal fluorescence microscopy with HC PL APO 63 × /1.40 OIL objective lens. Images were captured and processed with LAS-X.

For subcellular co-localization assay, after treatment with CQP probe, live parasite cultures were then incubated with the 300 nmol/L MitoTracker™ Deep Red FM (Invitrogen, Carlsbad, CA, USA) for 30 min at 37 °C. The subsequent operation was same as described above. The slides were imaged with Dragonfly 200 Spinning Disk Confocal Microscopy with HC PL APO 100 × /1.40 OIL objective lens. Images were captured with Fusion and processed with Imaris 9.3. The semi-quantitative analysis of the extent of co-localization was carried out using the JACop plugin in Image J software [27].

For co-localization with target proteins, after the click chemistry reaction, parasites were incubated with the indicated primary antibody and secondary fluorescent antibody (Additional file 3: Table S1) and imaged same as described in subcellular co-localization assay.

### Statistical analysis

All data were based on 3 independent biological replicates at least, and shown as mean ± standard error of the mean (SEM) unless stated otherwise. The statistical analysis was performed using one-way ANOVA variance tests in GraphPad Prism 8.3. Statistical significance was defined as a *P*-value of less than 0.05.

## Results

### Design and synthesis of a CQP

**Fig. 1:**
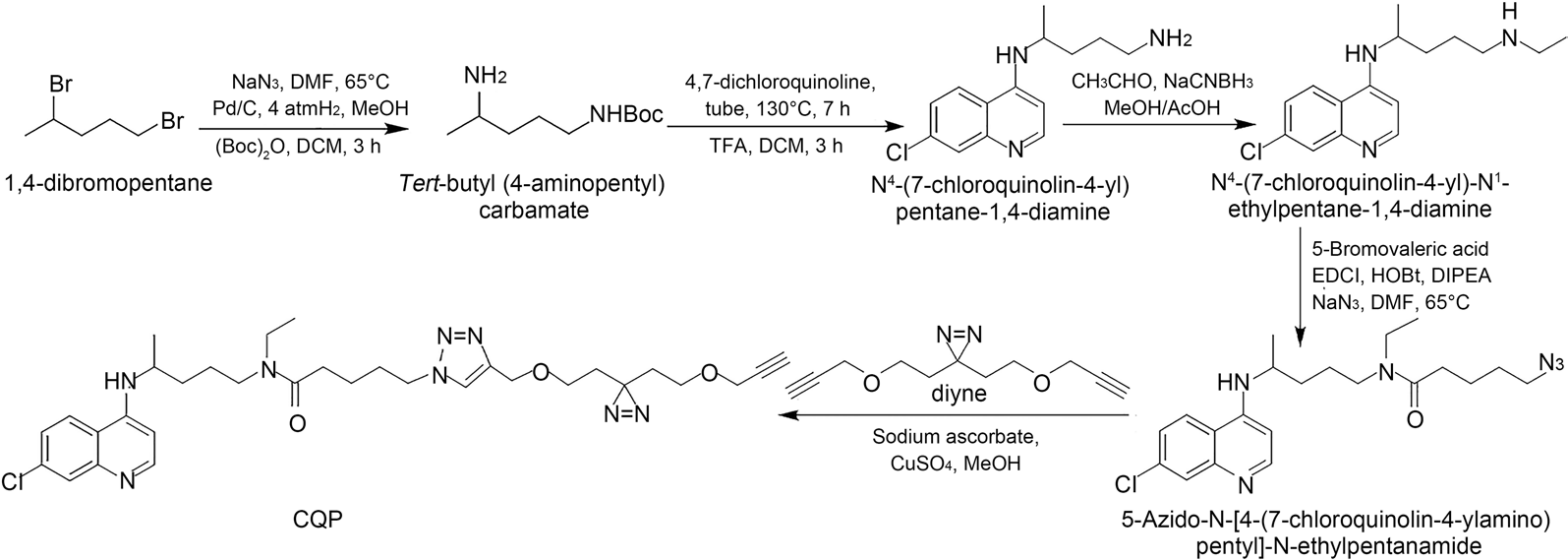
Synthesis scheme of chloroquine analogue activity probe CQP. DMF dimethyl formamide, DCM dichloromethane, TFA trifluoroacetic acid, Me methanol, Ac acetic acid, EDCI 1-(3-dimethylaminopropyl)-3-ethylcarbodiimide hydrochloride, HOBt 1-hydroxybenzotriazole, DIPEA N, N-diisopropylethylamine, CQP chloroquine analog probe

We used the strategy of photo-affinity labeling and click chemistry-based functionalization for CQP design [28–30]. Our synthesis of CQP began with the conversion of 1,4-dibromopentane to *tert*-butyl (4-aminopentyl) carbamate via a nucleophilic substitution, hydrogenation and Boc-protected sequence. The treatment of *tert*-butyl (4-aminopentyl) carbamate with 4,7-dichloroquinoline in DMSO at 130 °C followed by deprotection generated N^4^-(7-chloroquinolin-4-yl) pentane-1,4-diamine. Borch reduction of N^4^-(7-chloroquinolin-4-yl) pentane-1,4-diamine with acetaldehyde synthesized N^4^-(7-chloroquinolin-4-yl)-N^1^-ethylpentane-1,4-diamine, which was further subject to esterification and nucleophilic substitution with sodium azide to generate 5-Azido-N-[4-(7-chloroquinolin-4-ylamino) pentyl]-N-ethylpentanamide. The click reaction of azide 5-Azido-N-(4-(7-chloroquinolin-4-ylamino) pentyl)-N-ethylpentanamide with diyne finally produced CQP (Fig. 1). Notably, the CQP still retains the quinoline ring system, the chlorine at the 7-position and the terminal amino group that are indispensable for its antimalarial activity [31].

### Fluorescence labelling of CQP targets in parasites

**Fig. 2:**
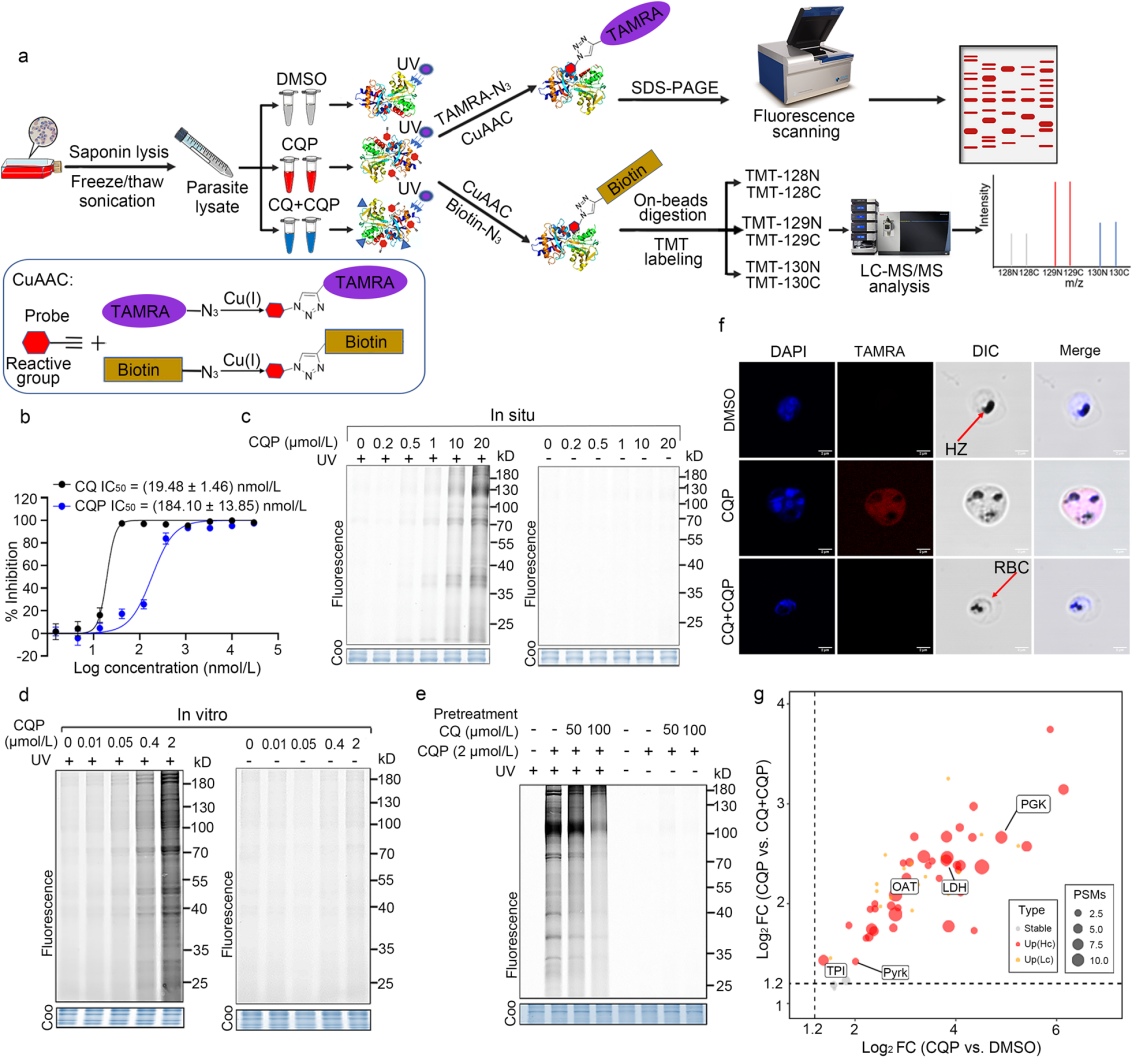
Identification of CQ target proteins by CQP through ABPP. **a** General workflow of photoaffinity CQP probe-mediated ABPP used to label and identify CQ target proteins. Parasite lysates were incubated with CQP probe, CQ or vehicle followed by irradiation with UV light (λ = 365 nm). The alkyne tag on the probe allows for the subsequent attachment of a fluorescent tag or biotin moiety through CuAAC-mediated click chemical reaction. The fluorescence labeling of parasite proteins is visualized by fluorescence scanning after resolving on SDS-PAGE gel. The pull-down of CQP-labeled proteins by biotin-streptavidin affinity purification is subject to TMT-based labeling and quantification on LC–MS/MS. The chemistry structures for fluorescent and biotin tags seen in Additional file 2: Fig. S1. **b** Determination of antimalarial activity of CQP and CQ in *P. falciparum* 3D7 strain. **c** In situ labeling of CQP in parasites living in infected red blood cells. The parasites were released and the lysates were prepared for click reaction. The amounts of proteins being labeled were in a dose-dependent manner after UV irradiation, while there was no labeling without UV irradiation. **d** In vitro labelling of CQP in extracted parasite lysates were consistent with the in situ labeling as shown in **c**. **e** Labeling of CQP-target proteins in parasite lysate can be competed with excess CQ. **f** Confocal microscopy showing the distribution of CQP (2 µmol/L) inside the *P. falciparum* 3D7 parasites, and eliminated mostly with excess CQ (20 µmol/L). **g** Scatter plot of 60 quantified proteins from CQP ABPP experiment. Each dot represents a quantified protein, with the x-axis represents the mean of log_2_ protein abundance difference between CQP and DMSO control group, while y-axis represents the mean of log_2_ difference between CQP and CQ + CQP group. Two biological replicates were included in the experiment. The dashed line represents the fold change cutoff criteria (> 1.2) used for hit selection. The gray points represent proteins with *P*_adj_ > 0.05. *P*_adj_ were generated from the empirical Bayes test model and adjusted using Benjamini–Hochberg. The proteins measured with only 1 peptide spectrum match were regarded as low-confidence, and colored in orange. The remaining 40 proteins were kept as high-confidence targets and colored in red and the dot size is proportional to the associated PSM numbers. DMSO dimethyl sulfoxide, CQ chloroquine, CQP chloroquine analog probe, TAMRA carboxytetramethylrhodamine, CuAAC copper-catalyzed azide-alkyne-cycloaddition, SDS-PAGE sodium lauryl sulfate–polyacrylamide gel electrophoresis, TMT tandem Mass Tag, LC–MS/MS liquid chromatography-tandem mass spectrometry, Coo comassie, DAPI 4',6-diamidino-2-phenylindole, HZ hemozoin, RBC red blood cell, FC fold change, PSMs peptide-spectrum matches

Firstly, we tested the antimalarial activity of the CQP in vitro on *P. falciparum* 3D7 strain (a well-characterized and widely-used CQ-sensitive strain [32–35]) and confirmed that the derivatized CQP probe killed 3D7 strain in the nanomole range, retaining the antimalarial activity of CQ (Fig. 2a). The photo-reactive group on CQP can generate highly active carbene chemical species, which would covalently cross-link with the target proteins upon UV irradiation (λ = 365 nm). Next, the samples can be further labeled with a TAMRA fluorescent tag through click chemistry reaction with TAMRA-azide reagent, as depicted in Fig. 2b. We went on to carry out fluorescence labeling experiments in situ (i.e., on live parasite in iRBCs) and in vitro (i.e., on parasite lysate), respectively. As shown in Fig. 2c, d, parasite proteins were labeled with TAMRA in a CQP dose-dependent manner after UV irradiation either in situ or in vitro. There was hardly any fluorescence labeling of parasite proteins without UV irradiation. Importantly, when pre-incubated with excess CQ, the fluorescence labeling was diminished (Fig. 2e), indicating that CQP and CQ share the same protein targets. Furthermore, the distribution of the CQP probe in the parasite was evaluated in a live cell imaging experiment with confocal microscopy and the results showed that the CQP probe can accumulate inside the parasites, and can be competed away by excess CQ (Fig. 2f), which is consistent with the competitive fluorescence labeling result on parasite lysate (Fig. 2e). In summary, the active photo-affinity CQP largely maintains the antimalarial activity and the intracellular accumulation characteristics of CQ. Meanwhile, it opens up the possibility to use this derivatized CQP probe to track the binding of CQ and target proteins in parasite.

### Identification of CQ targets with CQP using ABPP strategy

We decided to use this CQP to retrieve and identify the target proteins of CQ using ABPP strategy. The *P. falciparum* 3D7 parasite lysate was incubated with CQP or DMSO vehicle in the presence or absence of CQ, followed by UV irradiation and biotin moiety incorporation through click reaction. The CQP-binding targets were then retrieved by biotin-streptavidin affinity purification and identified by quantitative proteomics. The TMT-based multiplexing scheme was chosen for parallel analysis of samples from three different treatment conditions, in order to improve statistical power (Fig. 2a). We identified a total of 40 proteins that were specifically photo-crosslinked with CQP with high confidence, which was inhibited in the presence of excess CQ (Fig. 2g, Additional file 2: Fig. S2). The detailed information of these proteins was shown in Additional file 3: Table S2.

### Identification of CQ targets using MS-CETSA

**Fig. 3:**
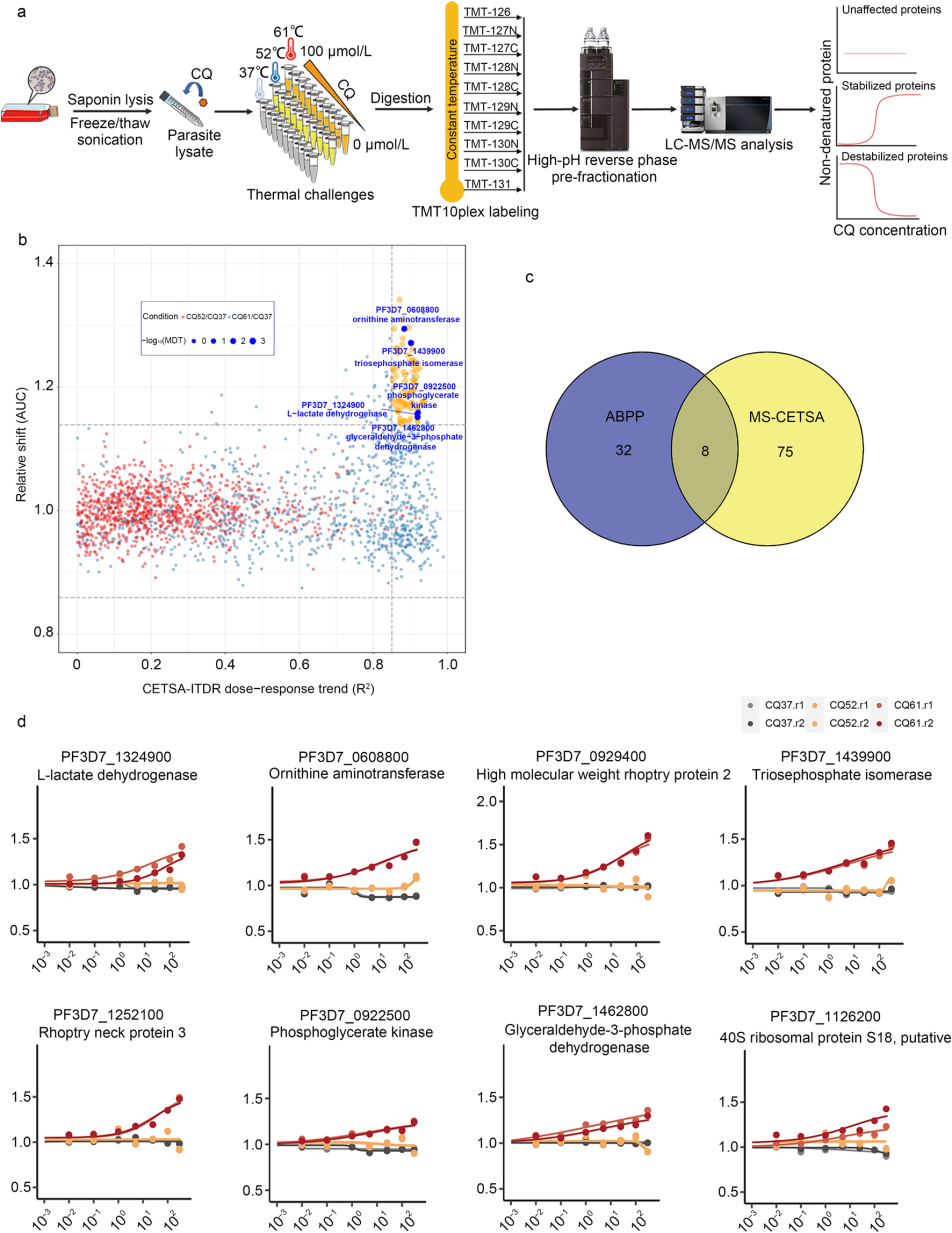
Identification of CQ target proteins through MS-CETSA. **a** General workflow of MS-CETSA used to identify CQ interacting protein. Parasite lysates were incubated with CQ at various concentrations, followed by heating under different temperature conditions. The remaining soluble proteins were collected for LysC/Trypsin digestion. The digested peptides are labeled by TMT10plex reagent and quantified by mass spectrometry. The profile of protein thermal stability along the CQ concentration gradient was generated. The proteins with significant thermal shifts were identified as the potential CQ-targeting proteins. **b** A R^2^-AUC plot showing the protein thermal stability shift of the whole *Plasmodium falciparum* proteome after incubation with 0–300 µmol/L CQ from the lysate ITDR MS-CETSA experiment. The potential hit proteins are in orange color, with the highlighted ones in dark blue color. **c** Venn diagram showing the overlap of target proteins identified in ABPP and MS-CETSA. **d** Thermal shift profile of the 8 overlap target proteins. CQ chloroquine, MDT minimum dose threshold, AUC area under the curve, ABPP activity-based protein profiling, MS-CETSA mass spectrometry-coupled cell thermal shift analysis, ITDR isothermal dose–response

We also used MS-CETSA, a recently developed proteome-wide and label-free target deconvolution method based on the biophysical principle that the thermal stability of target proteins tends to shift upon binding with drugs/compounds, to identify the target proteins of CQ (Fig. 3a) [26, 36]. By monitoring the impact of CQ in various concentrations (0–300 µmol/L) on the thermostability of the malarial parasite proteome under three different heating conditions (37 °C, 52 °C, 61 °C), we identified 83 candidate interacting proteins out of a total measurement of 3375 parasite proteins (Fig. 3b, Additional file 3: Table S3). Notably, we identified 8 proteins as the most potential hits which were commonly identified by both ABPP and MS-CETSA methods (Fig. 3c). Interestingly, the majority of them are involved in glycolysis and energy metabolism (Fig. 3d).

### Binding and functional verification of the CQ target proteins in vitro

**Fig. 4:**
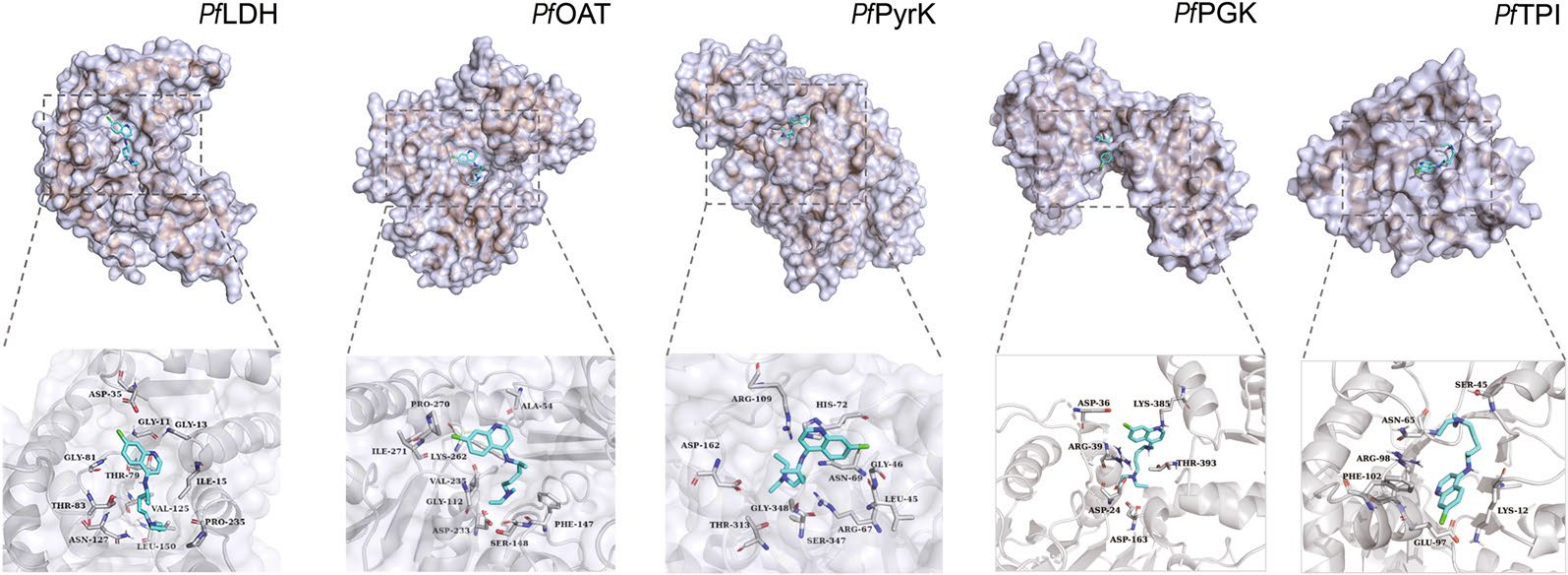
Docking simulation of CQ binding to *Pf*LDH, *Pf*OAT, *Pf*PyrK, *Pf*PGK and *Pf*TPI proteins, respectively. The calculated binding affinities (kcal/mol) are -6.2, -6.1, -6.5, -5.7 and -5.8 for *Pf*LDH, *Pf*OAT, *Pf*PyrK, *Pf*PGK and *Pf*TPI, respectively. *Pf Plasmodium falciparum*, LDH L-lactate dehydrogenase, OAT ornithine aminotransferase, PyrK pyruvate kinase, PGK phosphoglycerate kinase, TPI triosephosphate isomerase

We then went on to verify some of these interactions in detail, focusing on 5 enzymes involved in glycolysis and energy metabolism including *Pf*LDH (*PF3D7_1324900*), *Pf*OAT (*PF3D7_0608800*), *Pf*PyrK (*PF3D7_0626800*), *Pf*PGK (*PF3D7_0922500*) and *Pf*TPI (*PF3D7_1439900*). To start with, we retrieved the structures of these 5 proteins from PDB database and modelled the binding poses of CQ to them through molecular docking simulation (Fig. 4). Interestingly, in all cases, CQ binding was at or close to the pocket of substrate/active sites, implying the biological relevance of the binding events.

**Fig. 5:**
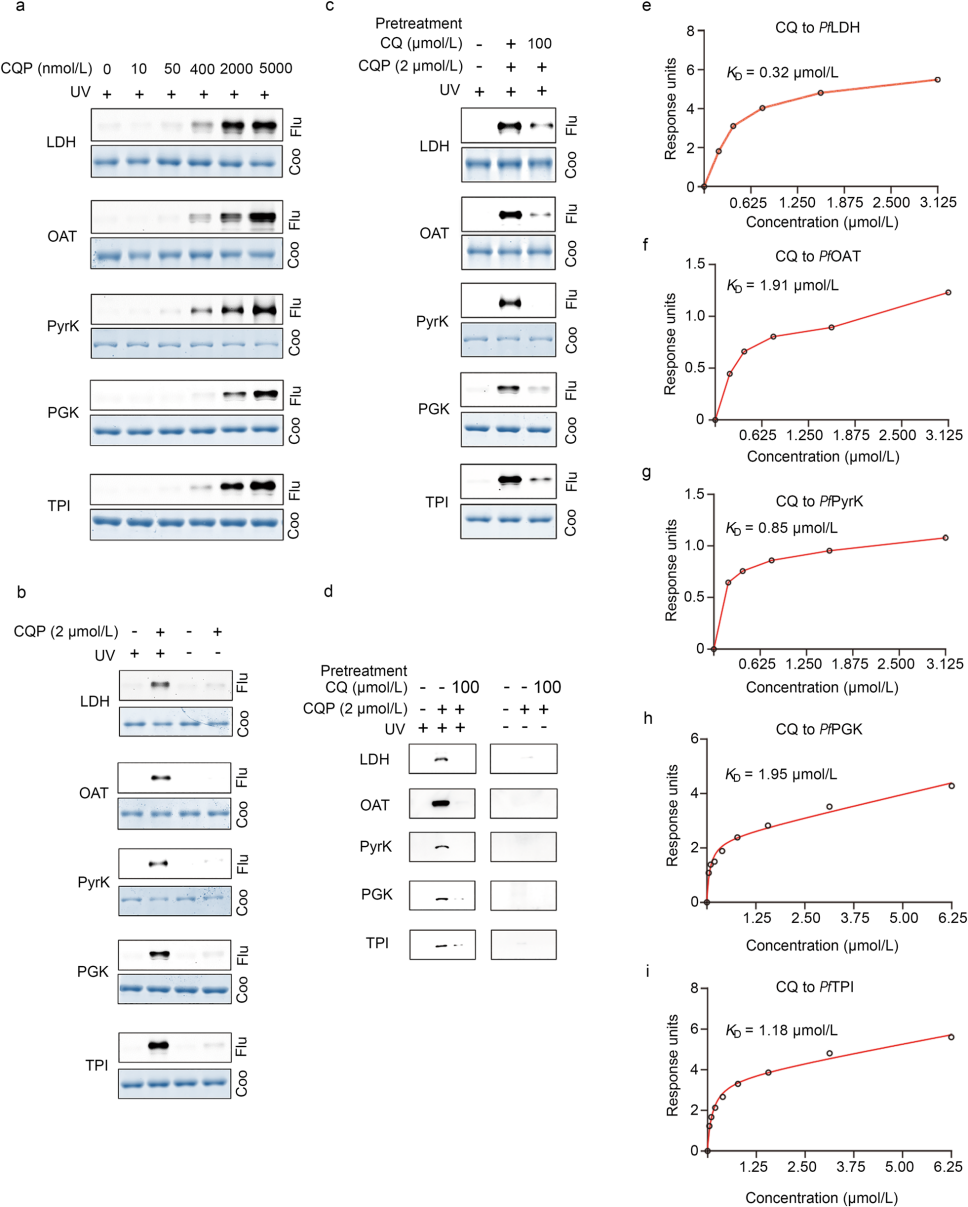
Binding verification of the CQ target proteins. **a** CQP specifically binds to recombinant *Pf*LDH, *Pf*OAT, *Pf*PyrK, *Pf*PGK*,* and *Pf*TPI proteins in a dose-dependent manner. **b** CQP labels recombinant parasite proteins only under UV irradiation. **c** Pre-treatment of excess CQ (50×) can compete with the CQP binding to the target proteins. **d** Validation of the specific targeting of CQP to the 5 target proteins in live parasites by western blot. **e-i** The determination of binding affinity of CQ with the 5 target proteins through SPR assay. CQ chloroquine, CQP chloroquine analog probe, Flu fluorescence, Coo coomassie, *Pf Plasmodium falciparum,* LDH L-lactate dehydrogenase, OAT ornithine aminotransferase, PyrK pyruvate kinase, PGK phosphoglycerate kinase, TPI triosephosphate isomerase

Next, the 5 proteins were recombinantly expressed in *Escherichia coli* and successfully purified. As expected, CQP was photo-crosslinked to these 5 recombinant proteins in a dose-dependent manner under UV irradiation (Fig. 5a, b), which was diminished by CQ pre-treatment (Fig. 5c). In order to confirm CQP targets to these 5 proteins in situ, the live iRBCs were incubated with the CQP probe and subject to UV irradiation. Parasite lysate was then prepared for click reaction-mediated biotin moiety incorporation and the pull-down experiment was carried out by incubation with Neutravidin beads. The bound proteins were eluted and detected by immunoblotting assay using target protein specific antibodies. The result confirmed that all the 5 proteins could be specifically targeted by CQP, and the interactions could be efficiently eliminated with CQ pre-treatment (Fig. 5d).

**Fig. 6:**
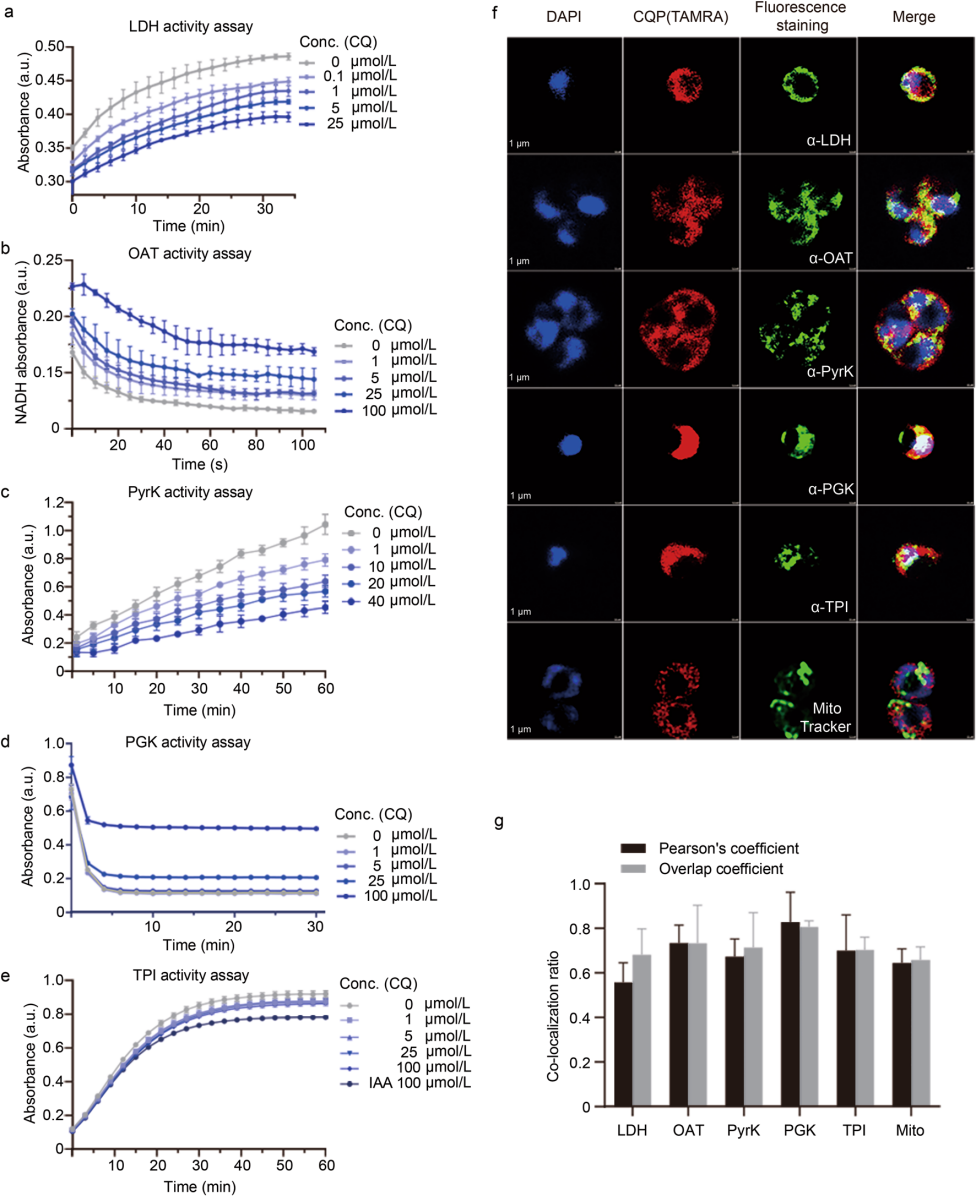
Functional verification of the CQ target proteins. CQ inhibits the enzymatic activities of the recombinant *Pf*LDH (**a**), *Pf*OAT (**b**), *Pf*PyrK (**c**), *Pf*PGK (**d**) and *Pf*TPI (**e**) proteins in vitro in a dose-dependent manner. **f** Immunofluorescence staining experiment of CQP (2 µmol/L) as well as the target proteins (by respective antibody) and the mitochondria (by MitoTracker, 300 nmol/L). **g** Co-localization analysis with Pearson's coefficient and Overlap coefficient. LDH L-lactate dehydrogenase, OAT ornithine aminotransferase, PyrK pyruvate kinase, PGK phosphoglycerate kinase, TPI triosephosphate isomerase, DAPI 4',6-diamidino-2-phenylindole, CQP chloroquine analog probe, TAMRA carboxytetramethylrhodamine, Mito mitochondria, Conc. concentration, a.u. absorbance unit

In addition, we measured the binding affinity between CQ and the 5 proteins by BIAcore SPR. All the measured binding events were at the level of low (*K*_CQ-*Pf*LDH_ = 0.32 µmol/L, *K*_CQ-*Pf*OAT_ = 1.91 µmol/L, *K*_CQ-*Pf*PyrK_ = 0.85 µmol/L, *K*_CQ-*Pf*PGK_ = 1.95 µmol/L, *K*_CQ-*Pf*TPI_ = 1.18 µmol/L, respectively) (Fig. 5e–i). More importantly, the catalytic activities of these enzymes could be largely inhibited by CQ in a dose-dependent manner (Fig. 6a–e). In summary, all these experimental evidences supported the functional binding of CQ to these 5 parasite enzymes involved in glycolysis and energy metabolism.

### CQ disrupts glycolysis and energy metabolism of parasites

Subsequently, we conducted Gene Ontology enrichment analysis on the target proteins commonly identified in ABPP and MS-CETSA strategy, and the result also suggested that CQ could disrupt the glycolytic and energy metabolic processes of parasite to exert antimalarial effects (Additional file 2: Figs. S3, S4). We also carried out immunofluorescence experiments with spinning disk confocal microscopy and confirmed that the CQP probe indeed co-localized with the relevant target proteins as well as the mitochondria inside parasite (Fig. 6f, g). Therefore, our results suggested a new working mechanism of CQ in parasite that CQ directly targets and modulates glycolysis and energy metabolism-related proteins to exert antimalarial effects.

## Discussion

In summary, we found that CQ could disrupt glycolysis and energy metabolism of malarial parasites through direct binding with some of the key enzymes, an overlooked mechanism that complements its known inhibitory effect of hemozoin formation.

It is well established that the malaria parasites mainly depend on glycolysis for energy supply in the erythrocytic stage [37]. Yayon et al. [38] reported the differential in vitro sensitivity of CQ at the different stages of the erythrocytic developmental cycle, in which parasites in the trophozoite and schizont stages were considerably more sensitive to CQ than in ring-stage. It is notable that the increase of CQ sensitivity was coincident with a marked rise in the rate of glucose consumption by iRBCs [38]. In addition, our work is consistent with the emerging view as well as research interest of targeting the mitochondria and the associated metabolic pathways in parasite for antimalarial strategy [39–41]. Using a fluorescent derivative of CQ probe, Woodland et al. [42] found that other than DV, CQ could associate with membranes including the parasite plasma membrane, the endoplasmic reticulum, and the mitochondria. Tewari et al. [33] integrated the changes of transcriptomic and metabolic data of *P. falciparum* to probe the alternative MoA of CQ. Among the identified set of core genes that are perturbed in response to CQ, several proteins in the glycolysis and energy metabolism processes were also present. All these emerging evidences support to our discovery of the directly binding of CQ to some of the key enzymes in glycolysis and energy metabolism pathway of malaria parasite. However, we note the study of CQ-resistant parasite strain is out of the scope of this work, future work should be on examining the involvement of these identified key enzymes in CQ-resistant parasites.

To the best of our knowledge, this is the first report of identifying the CQ antimalarial targets by a parallel usage of labelled (ABPP) and label-free (MS-CETSA) methods. ABPP and MS-CETSA use different working principles for target deconvolution. ABPP is based on the affinity association between the drug and its target, whereas the readout of CETSA is thermal stabilization/destabilization of proteins upon drug binding [43]. A distinctive feature in the experimental design of these two approaches is that a functional probe needs to be synthesized for ABPP but MS-CETSA uses the original unmodified compound [19]. Like many other high throughput strategies, each method has certain advantages and shortcomings. This is why we applied stringent criteria to select the commonly identified ones as CQ binding targets for downstream analysis. Even though we mainly focused on the shared protein targets from the two methods in this work, the other candidates identified by only one of the methods might also be worthy of detailed analysis in future studies. Of note, in the development of this paper, Wirjanata et al. [44] disclosed an MS-CETSA dataset with however limited proteome coverage, reporting a sole hit protein falcilysin (*PF3D7_1360800*). In our dataset, falcilysin as well as other hemoglobin proteolytic enzymes including plasmepsin II and plasmepsin IV also showed significant thermal shift. On the other hand, multidrug resistance protein 1 (*Pf*MDR1, *PF3D7_0523000*) was identified as an interacting hit protein in our ABPP dataset, which has been demonstrated in previous study [45, 46]. It should be noted that CQ and HCQ are also used to treat autoimmune disorders, such as rheumatoid arthritis and lupus erythematosus [16]. Therefore, the reagents and approaches developed in this study can guide the future research of the MoA of CQ and the like in those conditions.

## Conclusions

This work provides to a more complete understanding of the working mechanism of the antimalarial drug CQ. It is not our intention to challenge the classic view of CQ in inhibiting hemozoin formation. We instead attempted to unravel other overlooked mechanisms of CQ underlying its antimalarial activity. It is of great significance to alleviate the resistance of CQ in malaria treatment and expand the use of CQ for new indications. The 5 target proteins (*Pf*LDH, *Pf*OAT, *Pf*PyrK, *Pf*PGK and *Pf*TPI) we identified and verified are all important proteins related to glycolysis and energy metabolism of parasites, indicating that this is likely to be an important research direction related to antimalarial. In addition, this work established a new research strategy for the MoA study of other important but not-well-understood drugs.

## Supplementary Information

**Additional file 1**: Chemical synthesis schemes, NMR and MS Spectra, and supplementary methods.

**Additional file 2: Fig. S1**. Chemistry structures of TAMRA-N3 (A) and Biotin-N3 (B) of CuAAC-based click chemistry reaction. **Fig. S2** Heat map representation of the target proteins dataset identified by the CQP-based ABPP. The expression levels of all proteins are standardized to Z-score values. **Fig. S3** Gene Ontology (GO) analysis of the enriched biological process (BP) (**a**), cellular component (CC) (**b**) and molecular function (MF) (**c**) for chloroquine targets identified by CQP-based ABPP. **Fig. S4** GO enrichment analysis of biological process (BP) (**a**), cellular component (CC) (**b**) and molecular function (MF) (**c**) for chloroquine targets identified by MS-CETSA.

**Additional file 3: Table S1**. Antibodies used for Western blotting validation. **Table S2** Target proteins identified by CQP-based ABPP. **Table S3** Potential hits identified by MS-CETSA.

## Data Availability

The datasets used and/or analyzed during the present study are available from the corresponding author on reasonable request.

## Abbreviations

ABPP: Activity-based protein profiling
AUC: Area under the curve
CuAAC: Copper catalyzed azide alkyne cycloaddition
CQ: Chloroquine
CQP: Chloroquine analog probe
DMSO: Dimethyl sulfoxide
DV: Digestive vacuole
HCQ: Hydroxychloroquine
IC_50_: Half-maximal inhibitory concentration
iRBCs: Infected red blood cells
ITDR: Isothermal dose–response
LDH: Lactate dehydrogenase
MAD: Median absolute deviation
MCM: Malaria culture media
MoA: Mechanism of action
MS-CETSA: Mass spectrometry-coupled cellular thermal shift assay
OAT: Ornithine aminotransferase
PD: Proteome discoverer
PI: Protease inhibitor
PyrK: Pyruvate kinase
PGK: Phosphoglycerate kinase
RBCs: Red blood cells
RT: Room temperature
TAMRA-azide: Tetramethyl-6-carboxyrhodamine azide
TBTA: Tris[(1-benzyl-1H-1,2,3triazol-4-yl) methyl] amin
TCEP: Tris (2-carboxyethyl) phosphine
TPI: Triosephosphate isomerase
