# Supplementary material for: Identification of antimalarial targets of chloroquine by a combined deconvolution strategy of ABPP and MS-CETSA
Source: Mil Med Res. 2022 Jun 14;9:30. doi: 10.1186/s40779-022-00390-3 (PMC9195458; doi:10.1186/s40779-022-00390-3)
Supplement: Supplementary file 1 — Additional file 1: Chemical synthesis schemes, NMR and MS Spectra, and supplementary methods. [file 40779_2022_390_MOESM1_ESM.pdf]

Chemical synthesis schemes

All the reagents and solvents were purchased from Sigma-Aldrich, AK Scientific or Acros, and were used without further purification unless stated otherwise. Reactions were monitored by thin layer chromatography (TLC). Column chromatography was performed on silica gel of 200 - 300 mesh. All ^{1}H NMR (300 and 500 MHz), ^{13}C NMR (75 and 125 MHz) spectra were recorded. ^{1}H NMR Spectroscopy splitting patterns were designated as singlet (s), doublet (d), triplet (t), quartet (q). Splitting patterns that could not be interpreted or easily visualized were designated as multiplet (m).

***Tert*-butyl (4-aminopentyl) carbamate**

Sodium azide (4.3 g, 66 mmol) was added to a solution of 1,4-dibromopentane (5.1 g, 22 mmol) in dimethyl formamide (DMF, 50 ml). The mixture was stirred overnight at 60°C. After cooling to room temperature, the mixture was diluted by water (200 ml) and extracted with EtOAc (3 × 100 ml). The organic phase was dried over Na_2SO_4, filtered and concentrated under reduced pressure to afford the desired product as colorless oil (3.2 g, 95% yield). Mixture of 1,4-diazidopentane (3.2 g, 21 mmol) and Pd/C (400 mg) in MeOH (150 ml) was stirred for overnight under H_2 atmosphere at room temperature, before being filtered and the filtrate was concentrated to give pentane-1,4-diamine (2.1 g) which was used in next step without further purification. *Tert*-butyldicarbonate (4.4 g, 20.6 mmol) was added to a stirred solution of 1,4-pentandiamine (2.1 g, 20.6 mmol) in absolute EtOH (25 ml). The reaction mixture was refluxed 3 h, followed by removal of the volatiles in vacuum. Water was added and 2 mol/L HCl was added until pH = 3, followed by extraction with CH_2Cl_2. The aqueous phase was then made strongly alkaline by addition of aqueous NaOH (2 mol/L) and extracted with CH_2Cl_2. The combined organic extracts were dried over Na_2SO_4 and the solvent was evaporated under reduced pressure, the residue was purified by silica gel column chromatography, yielding product as colorless viscous oil (1.5 g, 36%).

N^4-(7-chloroquinolin-4-yl) pentane-1,4-diamine

A mixture of 4,7-dichloroquinoline (970 mg, 4.9 mmol) and *tert*-butyl (4-aminopentyl) carbamate (1.2 g, 5.9 mmol) in DMSO (5 ml) was heated at 130°C for 7 h in tube. The reaction mixture was cooled to room temperature, before being diluted by water and extracted with EtOAc. The organic layer was

washed with $NaHCO_3$, water and brine. Then the organic layer was dried over Na_2SO_4 and the solvent was evaporated under reduced pressure, the residue was purified by silica gel column chromatography, yielding product as colorless viscous oil (730 mg, 41%). A solution of *tert*-butyl {4-[(7-chloroquinolin-4-yl) amino] pentyl} carbamate (730 mg, 2.0 mmol) in TFA/CH_2Cl_2 (v: v; 1: 10), was stirred at room temperature for 3 h. The solvent was evaporated under reduced pressure and the remaining residue was treated with CH_2Cl_2/2.5 mol/L NaOH. The organic layer was evaporated under reduced pressure and was further purified by silica gel column chromatography, yielding product as colorless powder (394.5 mg, 75%).

N^4-(7-chloroquinolin-4-yl)-N^1-ethylpentane-1,4-diamine

N^4-(7-chloroquinolin-4-yl) pentane-1,4-diamine (394.5 mg, 1.5 mmol) and acetaldehyde (165 mg, 1.5 mmol, 40%) were dissolved in MeOH (20 ml), AcOH (1 ml) was added, the mixture was stirred at room temperature for 3 h. Then, $NaCNBH_3$ (283 mg, 4.5 mmol) was added and stirring was continued for another 1 h. The solvent was removed under reduced pressure and the residue was purified by silica gel column chromatography, yielding crude product as colorless powder (280 mg).

5-Azido-N-[4-(7-chloroquinolin-4-ylamino) pentyl]-N-ethylpentanamide

To a solution of N^4-(7-chloroquinolin-4-yl)-N^1-ethylpentane-1,4-diamine (280 mg, crude) and 5-bromopentanoic acid (180 mg, 1 mmol) in DMF (10 ml), EDCI (191 mg, 1 mmol), DIPEA (260 mg, 2 mmol) and 1-hydroxybenzotriazole (HOBt, 135 mg, 1 mmol) were added, the mixture was stirred at room temperature for 3 h. Then diluted by water and extracted with EtOAc. The organic layer was washed with water and the solvent was evaporated under reduced pressure, the residue was purified by silica gel column chromatography, yielding product as light yellow solid (170 mg). A mixture of bromo-N-[4-(7-chloroquinolin-4-ylamino) pentyl]-N-ethylpentanamide (170 mg, 0.38 mmol) and NaN_3 (36 mg, 0.56 mmol) in DMF (5 ml) was heated at 65°C for overnight. The reaction mixture was cooled to room temperature, before being diluted by water and extracted with EtOAc. The organic layer was washed with $NaHCO_3$, water and brine. Then the organic layer was dried over Na_2SO_4 and the solvent was evaporated under reduced pressure, the residue was purified by prep-HPLC to give the final product 33 mg as colorless oil. ^1H NMR (300 MHz, CD_3OD) δ 8.33-8.30 (m, 1H), 8.10-8.05 (m,

1H), 7.75 (s, 1H), 7.39-7.36 (m, 1H), 6.52-6.49 (m, 1H), 4.61-4.45 (m, 1H), 4.08-3.95 (m, 1H), 3.40-3.34 (m, 2H), 3.24-3.20 (m, 2H), 2.41-2.38 (m, 2H), 1.70-1.54 (m, 9H), 1.25-1.09 (m, 7H). LC-MS m/z: $[M+H]^+$ Calcd for $C_{21}H_{30}ClN_6O$ 417.21; Found 417.20.

CQP

To a solution of 5-azido-N-(4-(7-chloroquinolin-4-ylamino) pentyl)-N-ethylpentanamide (20.8 mg, 0.05 mmol), diyne (51.5 mg, 0.25 mmol), $CuSO_4$ (1.6 mg, 0.01 mmol) and sodium ascorbate (19.8 mg, 0.1 mmol) in 2 ml of MeOH were added. The reaction mixture was stirred at room temperature for 20 h and followed by ether (10 ml) dilution, was then washed with brine (5 ml). The resulting organic layer was concentrated under reduced pressure. Purification of the crude product by flash column chromatography, gave the final product CQP as colorless oil (13.6 mg, 43% yield). ^1H NMR (500 MHz, dimethyl sulfoxide-d_6), δ 8.50-8.47 (m, 2H), 8.06 (d, J = 5 Hz, 1H), 7.93 (s, 1H), 7.67-7.63 (m, 1H), 6.73-6.70 (m, 1H), 4.4 (s, 2H), 4.36-4.33 (m, 2H), 4.06 (d, J = 5Hz, 2H), 3.43-3.33 (m, 7H), 3.27-3.24 (m, 5H), 2.33-2.28 (m, 1H), 1.84-1.79 (m, 2H), 1.61-1.41 (m, 10H). 1.12-0.98 (m, 6H). HRMS m/z: $[M+H]^+$ Calcd for $C_{32}H_{44}ClN_8O_3$ 623.3219; Found 623.3221.

NMR and MS Spectra

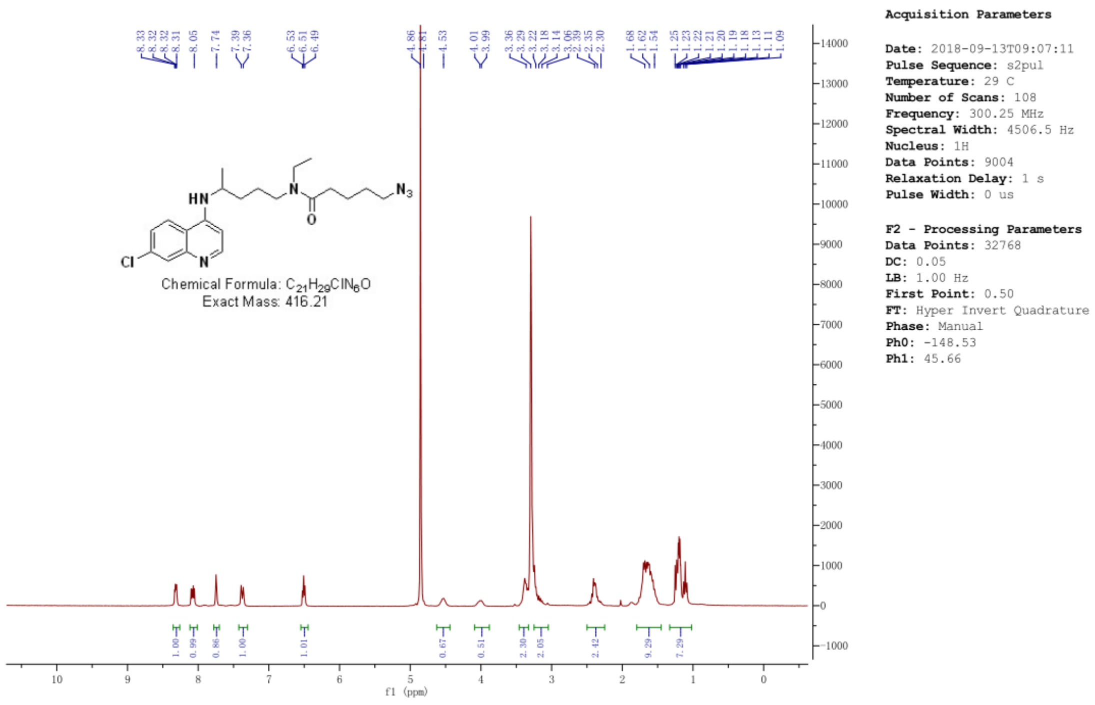

^{1}H-NMR of 5-Azido-N-[4-(7-chloroquinolin-4-ylamino) pentyl]-N-ethylpentanamide

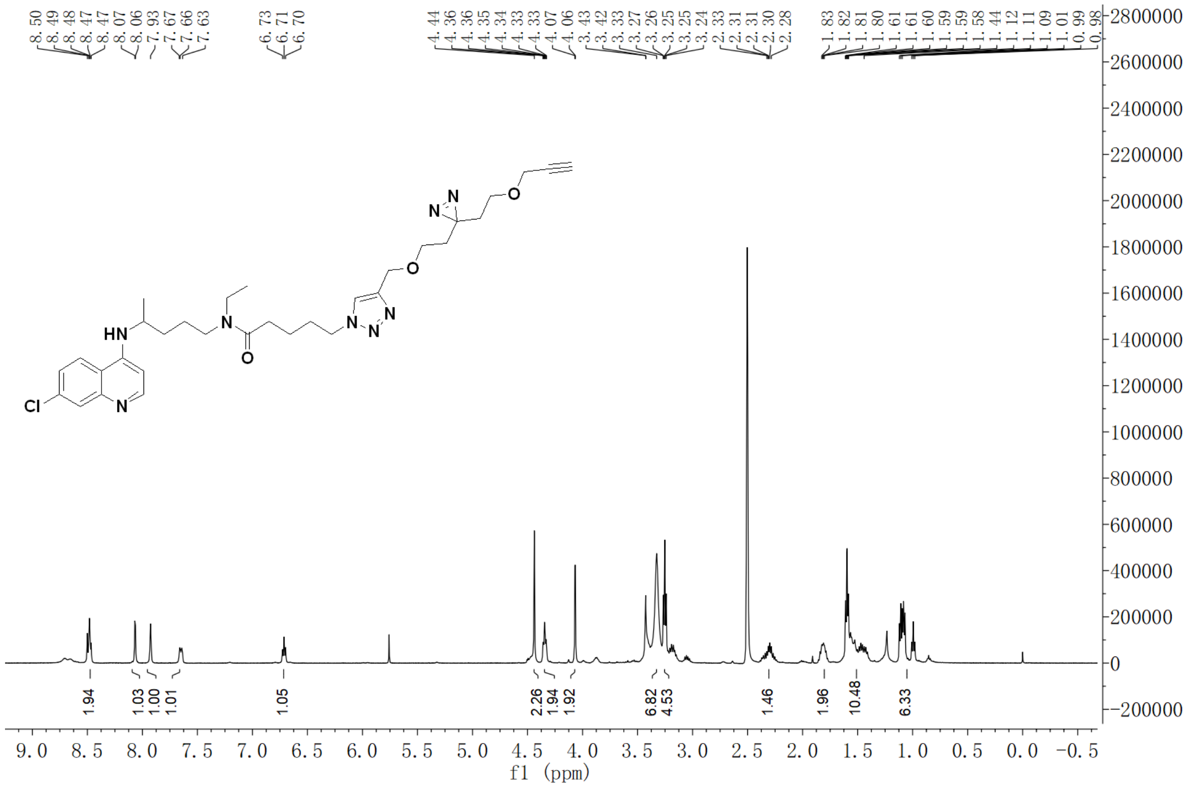

^{1}H-NMR of CQP

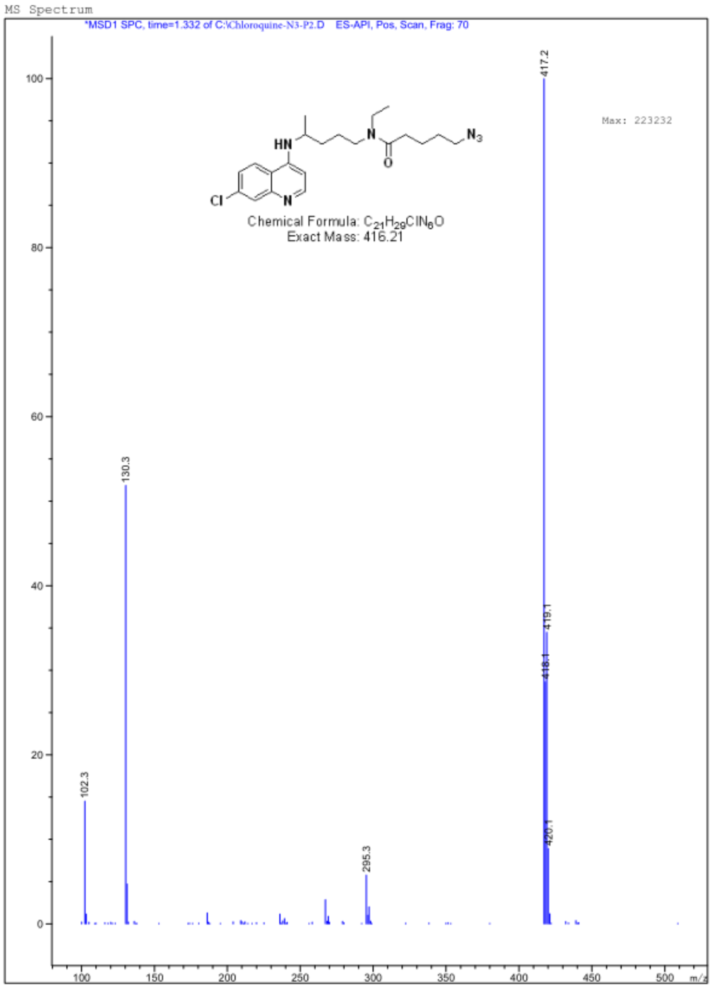

LC-MS of 5-Azido-N-(4-(7-chloroquinolin-4-ylamino) pentyl)-N-ethylpentanamide

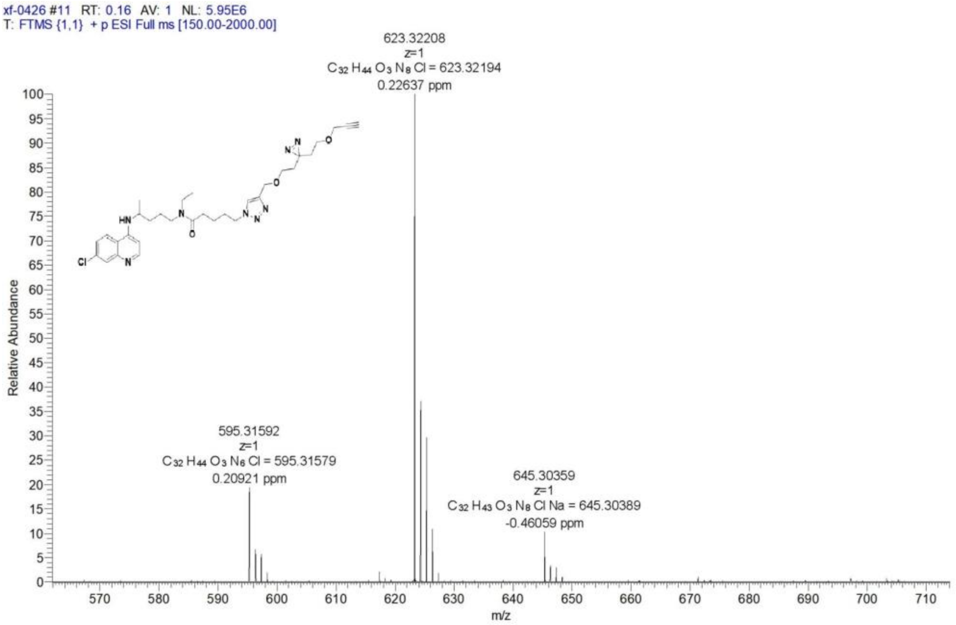

HRMS of CQP

Supplementary methods

1. L-lactate dehydrogenase (LDH) activity assay

The LDH Activity Assay Kit (Beyotime, Beijing, China) was used to monitor the activity of *Pf*LDH protein. Purified *Pf*LDH (0.5 μg) was first incubated with different concentrations of CQ (0 – 25 μmol/L) at 28°C for 1 h. Afterwards, the mixture was transferred to a 96-well plate, and the LDH detection buffer was then added, followed by continuous detection of absorbance at 490 nm with a multimode plate reader (PerkinElmer, Waltham, Mass, USA). The reaction buffer with CQ but no LDH was used as negative control. The measurements were carried out in triplicate.

2. Ornithine aminotransferase (OAT) activity assay

The δ-OAT Activity Detection Assay Kit (Solarbio, China) was applied to monitor the activity of *Pf*OAT protein. Purified *Pf*OAT protein (0.2 μg) was first incubated with different concentrations of CQ (0 – 100 μmol/L) at 28°C for 1 h. The resulting mixture was then transferred to a 96-well plate, and the substrate nicotinamide adenine dinucleotide (NADH) and OAT reaction buffer were then added, followed by continuous detection of NADH absorbance at 340 nm. The reaction buffer with CQ but no *Pf*OAT was used as negative control. The measurements were carried out in triplicate.

3. Pyruvate kinase (PyrK) activity assay

The PyrK Activity Assay Kit (Abcam, England) was used to monitor the activity of *Pf*PyrK. Purified protein (0.5 μg) was first incubated with different concentrations of CQ (0 – 40 μmol/L) at 28°C for 1 h. The resulting mixture was then transferred to a 96-well plate, the assay buffer and substrate mix were added followed by continuous detection of absorbance at 570 nm. The reaction buffer with CQ but no PyrK was used as negative control. The measurements were carried out in triplicate.

4. Phosphoglycerate kinase (PGK) activity assay

The Activity Assay Kit (Abcam, Cambridge, UK) was applied to monitor the activity of *Pf*PGK protein. Purified *Pf*PGK protein (0.5 μg) was first incubated with different concentrations of CQ (0 – 100 μmol/L) at 28°C for 1 h. The resulting mixture was then transferred to a 96-well plate, and the reaction mix buffer (including *Pf*PGK substrate and developer, adenosine triphosphate and NADH) was then

added, followed by continuous detection of NADH absorbance at 340 nm. The reaction buffer with CQ but no *Pf*PGK was used as negative control. The measurements were carried out in triplicate.

5. Triose phosphate isomerase (TPI) activity assay

The TPI Activity Colorimetric Assay Kit (BioVision, San Francisco, USA) was applied to monitor the activity of *Pf*TPI protein. Purified *Pf*TPI protein (0.5 μg) was first incubated with different concentrations of CQ (0 – 100 μmol/L) at 28°C for 1 h. The resulting mixture was then transformed into a 96-well plate, and the reaction mix buffer (including TPI substrate, developer and enzyme mix) was then added, followed by continuous detection of absorbance at 450 nm with a multimode plate reader (PerkinElmer, Waltham, Mass, USA). The reaction buffer with CQ but no *Pf*TPI was used as negative control. The measurements were carried out in triplicate.

6. Surface plasmon resonance (SPR) assay

Multi-cycle kinetics were run on a Biacore T200 instrument to investigate the binding kinetics of CQ against the potential target proteins. The flow cell temperature was 25°C. Normalized steady-state binding curves from SPR showing binding of CQ to proteins immobilized on the CM5 Series S sensor chip (Cytiva Life Sciences, CT, USA) surface. Different dilutions of CQ were flowed over the sensor chip surface at 30 μl/min for 60 s followed by 120 s of dissociation flow. Data from single cycle kinetics were analyzed using BIAevaluation software. All curves base lines were adjusted to zero, and injection start times were aligned. The reference sensorgrams was subtracted from the experimental sensorgrams to generate curves representing specific binding. A 1:1 binding model (Langmuir) was used to evaluate the binding kinetics to obtain the association rate constant (k_a) and dissociation rate constant (k_d). Binding affinity (K_D) was estimated based on the concentration dependence of the observed steady-state responses.

7. Molecular docking model

The 3D structure file of CQ was downloaded from PubChem. The structures of *Pf*LDH (PDB: 3ZH2), *Pf*PyrK (PDB: 6KSH), *Pf*OAT (PDB: 1LX9), *Pf*PGK (PDB: 3OZ7) and *Pf*TPI (PDB: 1M7P) were downloaded from RCSB PDB. The AutoDock tool was used to handle these structure files and transfer

their format into PDBQT. Then, the AutoDock Vina (v.1.1.2) was employed for docking CQ to the substrate binding site of each target protein. The grid box was centered to each binding sites with the same size (size_x = 30, size_y = 30, size_z = 30). The exhaustiveness parameter was set to 10 to find a better binding pose with lower affinity score.
