# Supplementary material for: Identification of antimalarial targets of chloroquine by a combined deconvolution strategy of ABPP and MS-CETSA
Source: Mil Med Res. 2022 Jun 14;9:30. doi: 10.1186/s40779-022-00390-3 (PMC9195458; doi:10.1186/s40779-022-00390-3)
Supplement: Supplementary file 2 — Additional file 2: Fig. S1. Chemistry structures of TAMRA-N3 (A) and Biotin-N3 (B) of CuAAC-based click chemistry reaction. Fig. S2 Heat map representation of the target proteins dataset identified by the CQP-based ABPP. The expression levels of all proteins are standardized to Z-score values. Fig. S3 Gene Ontology (GO) analysis of the enriched biological process (BP) (a), cellular component (CC) (b) and molecular function (MF) (c) for chloroquine targets identified by CQP-based ABPP. Fig. S4 GO enrichment analysis of biological process (BP) (a), cellular component (CC) (b) and molecular function (MF) (c) for chloroquine targets identified by MS-CETSA. [file 40779_2022_390_MOESM2_ESM.pdf]

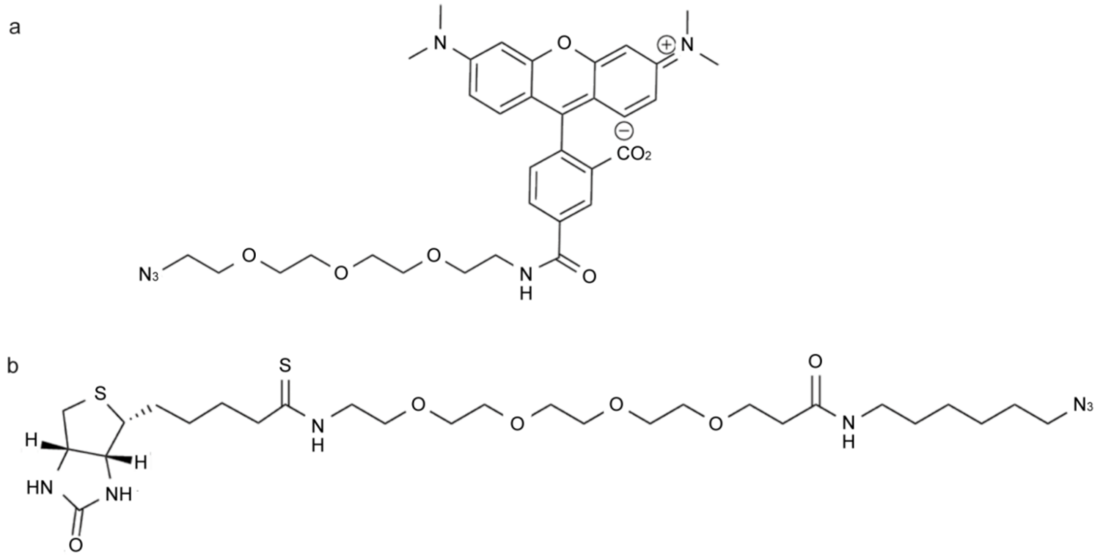

Fig. S1 Chemistry structures of TAMRA-N_3 (**a**) and Biotin-N_3 (**b**) of CuAAC-based click chemistry reaction

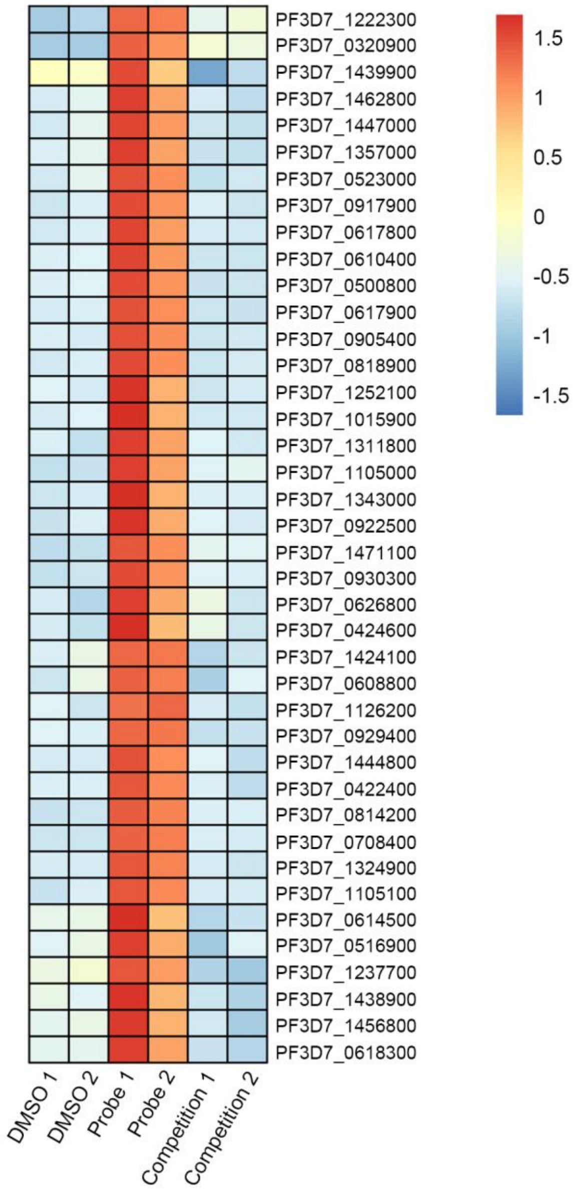

Fig. S2 Heat map representation of the target proteins dataset identified by the CQP-based ABPP. The expression levels of all proteins are standardized to Z-score values. CQP chloroquine analog probe, ABPP activity-based protein profiling

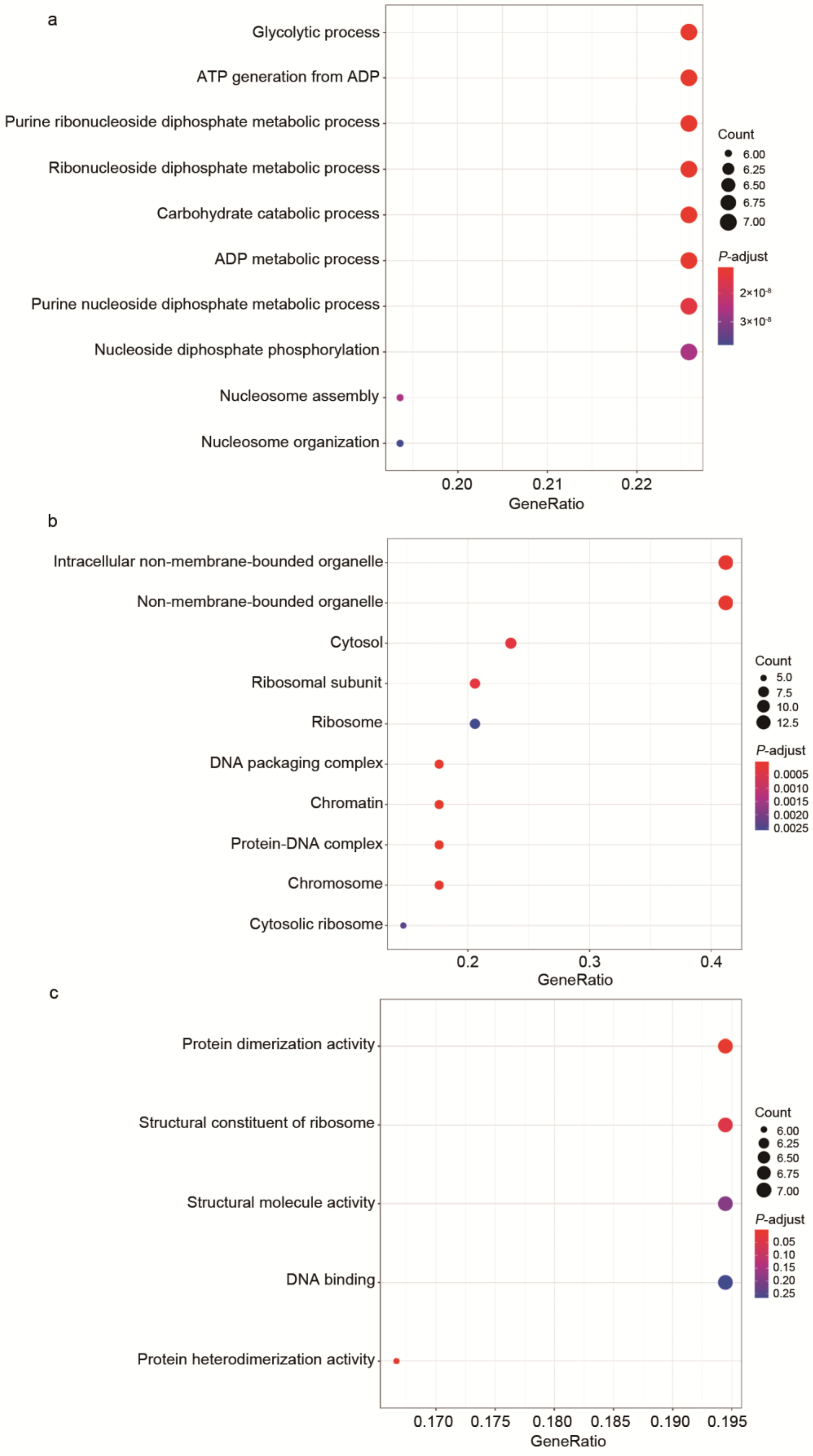

Fig. S3 Gene ontology (GO) analysis of the enriched biological process (BP) (**a**), cellular component (CC) (**b**) and molecular function (MF) (**c**) for chloroquine targets identified by CQP-based ABPP. CQP chloroquine analog probe, ABPP activity-based protein profiling

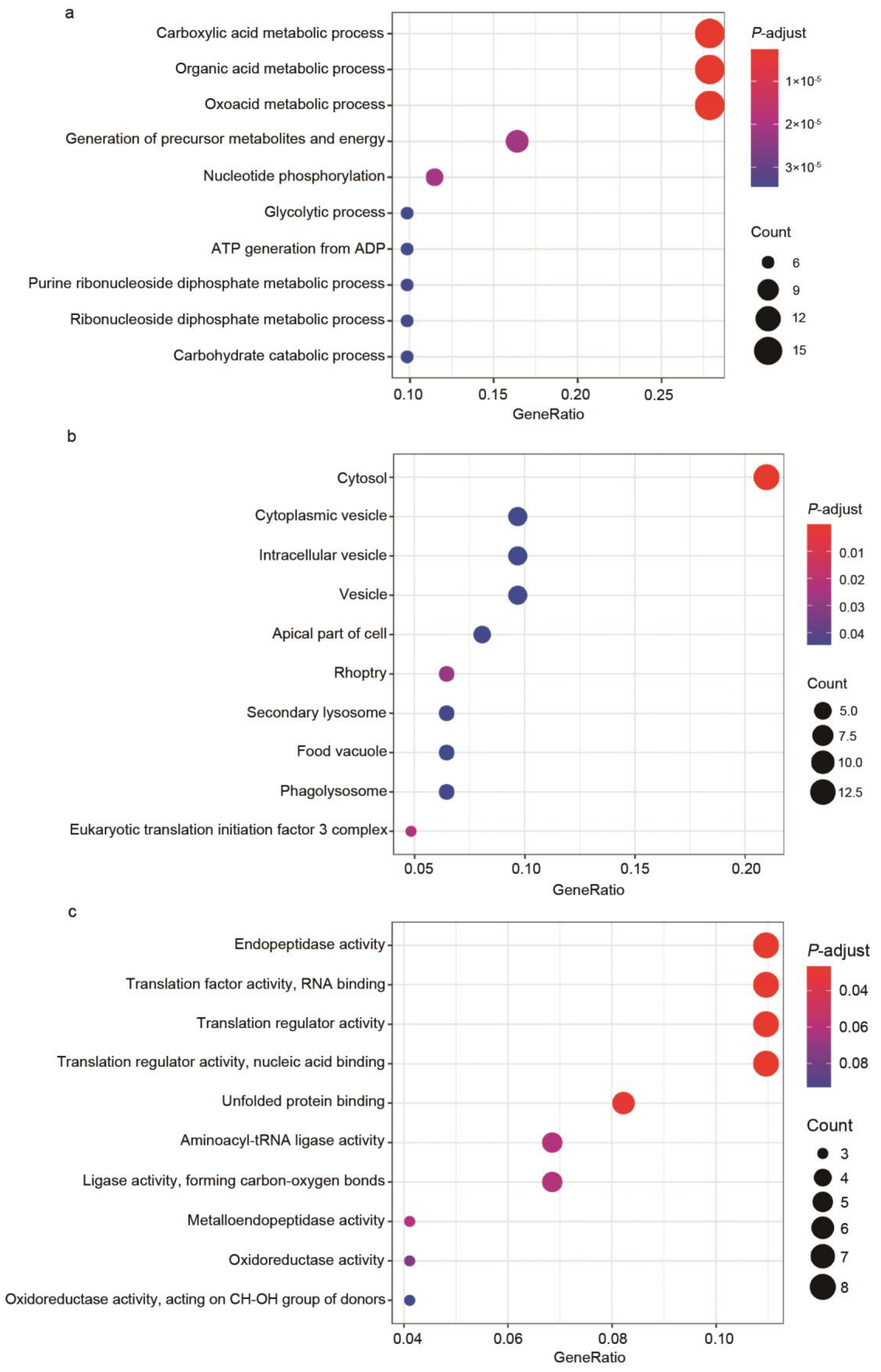

Fig. S4 GO enrichment analysis of biological process (BP) (**a**), cellular component (CC) (**b**) and molecular function (MF) (**c**) for chloroquine targets identified by MS-CETSA. MS-CETSA mass spectrometry-coupled cell thermal shift analysis
