# Supplementary material for: Identification of antimalarial targets of chloroquine by a combined deconvolution strategy of ABPP and MS-CETSA
Source: Mil Med Res. 2022 Jun 14;9:30. doi: 10.1186/s40779-022-00390-3 (PMC9195458; doi:10.1186/s40779-022-00390-3)
Supplement: Supplementary file 3 — Additional file 3: Table S1. Antibodies used for Western blotting validation. Table S2 Target proteins identified by CQP-based ABPP. Table S3 Potential hits identified by MS-CETSA. [file 40779_2022_390_MOESM3_ESM.pdf]

Table S1 Antibodies used for Western blotting validation

Product name	Brand	Cat.No.
Anti-ornithine aminotransferase antibody	Abcam	ab137679
PKM1/2 (C5E6) Rabbit mAb	Cell Signaling Technology	#3106
LDHA-Specific Polyclonal antibody	Proteintech	19987-1-AP
TPI1 Polyclonal antibody	Proteintech	10713-1-AP
PGK1 Polyclonal antibody	Proteintech	17811-1-AP
Anti-rabbit IgG, HRP-linked antibody	Cell Signaling Technology	7074S
Alexa Fluor® 488 Secondary antibody	Abcam	Ab150077

PKM1/2 pyruvate kinase M1/2, *LDHA* lactate dehydrogenase A, *TPI1* triosephosphate isomerase 1, *PGK1* phosphoglycerate kinase 1, *IgG* immunoglobulin G, *HRP* horseradish peroxidase

Table S2 Target proteins identified by CQP-based ABPP

No.	Accession	Description	MW (kD)	-logFC
1	PF3D7_0320900	Histone H2A.Z	16.45	2.27
2	PF3D7_0422400	40S ribosomal protein S19	19.72	2.58
3	PF3D7_0424600	Plasmodium exported protein (PHISTb)	35.96	1.62
4	PF3D7_0500800	Mature parasite-infected erythrocyte surface antigen	168.29	3.02
5	PF3D7_0516900	60S ribosomal protein L2	28	2.32
6	PF3D7_0523000	Multidrug resistance protein 1	162.25	3.59
7	PF3D7_0608800	Ornithine aminotransferase	46.06	2.26
8	PF3D7_0610400	Histone H3	15.45	2.67
9	PF3D7_0614500	60S ribosomal protein L19	21.58	1.62
10	PF3D7_0617800	Histone H2A	14.12	3.35
11	PF3D7_0617900	Histone H3 variant	15.44	3.25
12	PF3D7_0618300	60S ribosomal protein L27a, putative	16.72	1.66
13	PF3D7_0626800	Pyruvate kinase	55.66	0.89
14	PF3D7_0708400	Heat shock protein 90	86.17	2.96
15	PF3D7_0814200	DNA/RNA-binding protein Alba 1	27.26	2.89
16	PF3D7_0818900	Heat shock protein 70	73.92	2.88
17	PF3D7_0905400	High molecular weight rhoptry protein 3	104.86	5.03
18	PF3D7_0917900	Heat shock protein 70	72.39	1.8
19	PF3D7_0922500	Phosphoglycerate kinase	45.43	3.74
20	PF3D7_0929400	High molecular weight rhoptry protein 2	162.67	2.6
21	PF3D7_0930300	Merozoite surface protein 1	195.73	3.2
22	PF3D7_1015900	Enolase	48.68	1.9
23	PF3D7_1105000	Histone H4	11.46	3.95
24	PF3D7_1105100	Histone H2B	13.13	2.61
25	PF3D7_1126200	40S ribosomal protein S18, putative	17.89	1.28
26	PF3D7_1222300	Endoplasmin, putative	95.02	2.13

27	PF3D7_1237700	Conserved protein, unknown function	23.66	1.27
28	PF3D7_1252100	Rhoptry neck protein 3	263.16	1.42
29	PF3D7_1311800	M1-family alanyl aminopeptidase	126.06	2.92
30	PF3D7_1324900	L-lactate dehydrogenase	34.11	2.83
31	PF3D7_1343000	Phosphoethanolamine N-methyltransferase	31.04	4.92
32	PF3D7_1357000	Elongation factor 1-alpha	48.96	2
33	PF3D7_1424100	60S ribosomal protein L5, putative	34	2.4
34	PF3D7_1438900	Thioredoxin peroxidase 1	21.87	2.61
35	PF3D7_1439900	Triosephosphate isomerase	27.94	0.72
36	PF3D7_1444800	Fructose-bisphosphate aldolase	40.11	1.31
37	PF3D7_1447000	40S ribosomal protein S5	29.96	1.85
38	PF3D7_1456800	V-type H^+-translocating pyrophosphatase, putative	76.42	1.7
39	PF3D7_1462800	Glyceraldehyde-3-phosphate dehydrogenase	36.64	1.43
40	PF3D7_1471100	Exported protein 2	33.41	2.69

CQP chloroquine analog probe, *ABPP* activity-based protein profiling

Table S3 Potential hits identified by MS-CETSA

No.	Accession	Description	PSMs	MDT	R^2
1	PF3D7_0102900	Aspartate – tRNA ligase	24	0.022	0.880
2	PF3D7_0105200	Heptatricopeptide repeat and RAP domain-containing protein, putative	15	0.044	0.913
3	PF3D7_0212300	Eukaryotic peptide chain release factor subunit 1, putative	10	0.084	0.917
4	PF3D7_0214000	T-complex protein 1 subunit theta	16	0.000	0.931
5	PF3D7_0217900	Thioesterase/thiol ester dehydrase-isomerase, putative	7	0.017	0.914
6	PF3D7_0306800	T-complex protein 1 subunit beta	26	0.172	0.969
7	PF3D7_0314000	HSP20-like chaperone, putative	18	0.381	0.883
8	PF3D7_0315100	Eukaryotic translation initiation factor 4E	6	0.033	0.906
9	PF3D7_0316800	40S ribosomal protein S15A, putative	16	0.001	0.863
10	PF3D7_0320300	T-complex protein 1 subunit epsilon	18	0.000	0.919
11	PF3D7_0320700	Signal peptidase complex subunit 2	8	0.028	0.845
12	PF3D7_0418200	Eukaryotic translation initiation factor 3 subunit M, putative	17	0.006	0.871
13	PF3D7_0501500	Rhoptry-associated protein 3	35	0.030	0.914
14	PF3D7_0504600	2-oxoisovalerate dehydrogenase subunit beta, mitochondrial, putative	8	0.072	0.875
15	PF3D7_0523100	Mitochondrial-processing peptidase subunit alpha, putative	11	0.023	0.924
16	PF3D7_0524000	Karyopherin beta	75	0.000	0.895
17	PF3D7_0525100	Acyl-CoA synthetase	48	0.051	0.843
18	PF3D7_0529000	Conserved Plasmodium protein, unknown function	8	0.072	0.940
19	PF3D7_0606800	VFT protein	35	0.376	0.890
20	PF3D7_0608700	T-complex protein 1 subunit zeta	23	0.004	0.881
21	PF3D7_0608800	Ornithine aminotransferase	68	0.018	0.874
22	PF3D7_0624000	Hexokinase	31	0.007	0.843
23	PF3D7_0716800	Eukaryotic translation initiation factor 3 subunit I, putative	24	0.005	0.895
24	PF3D7_0802000	Glutamate dehydrogenase, putative	20	0.019	0.961

25	PF3D7_0807300	Ras-related protein Rab-18	24	0.301	0.862
26	PF3D7_0811200	ER membrane protein complex subunit 1, putative	18	0.004	0.885
27	PF3D7_0816600	Chaperone protein ClpB1	44	0.027	0.897
28	PF3D7_0820700	2-oxoglutarate dehydrogenase E1 component	9	0.004	0.812
29	PF3D7_0822600	Protein transport protein SEC23	13	0.004	0.918
30	PF3D7_0903400	ATP-dependent RNA helicase DDX60, putative	14	0.012	0.934
31	PF3D7_0904800	Replication protein A1, small fragment	23	0.022	0.862
32	PF3D7_0918300	Eukaryotic translation initiation factor 3 subunit F, putative	13	0.001	0.899
33	PF3D7_0920800	Inosine-5'-monophosphate dehydrogenase	17	0.031	0.859
34	PF3D7_0922500	Phosphoglycerate kinase	89	0.130	0.928
35	PF3D7_0927300	Fumarate hydratase	20	0.110	0.882
36	PF3D7_0929400	High molecular weight rhoptry protein 2	258	0.015	0.923
37	PF3D7_0933600	Mitochondrial-processing peptidase subunit beta, putative	15	0.005	0.830
38	PF3D7_0934500	V-type proton ATPase subunit E, putative	12	0.007	0.935
39	PF3D7_1008900	Adenylate kinase	38	1.831	0.911
40	PF3D7_1010700	Dolichyl-phosphate-mannose-protein mannosyltransferase, putative	16	0.039	0.860
41	PF3D7_1015200	Cysteine – tRNA ligase	10	0.007	0.934
42	PF3D7_1017900	26S proteasome regulatory subunit p55, putative	15	0.042	0.895
43	PF3D7_1033700	Bromodomain protein 1	16	0.009	0.934
44	PF3D7_1034900	Methionine – tRNA ligase	28	0.002	0.875
45	PF3D7_1103600	Actin-like protein, putative	8	0.001	0.904
46	PF3D7_1104400	Thioredoxin-like mero protein	61	0.018	0.863
47	PF3D7_1106000	RuvB-like helicase 2	7	0.063	0.849
48	PF3D7_1108400	Casein kinase 2, alpha subunit	10	0.000	0.893
49	PF3D7_1117300	Conserved protein, unknown function	15	2.015	0.947
50	PF3D7_1120100	Phosphoglycerate mutase, putative	24	0.094	0.915
51	PF3D7_1120500	tRNA nucleotidyltransferase, putative	9	0.019	0.899
52	PF3D7_1124600	Ethanolamine kinase	6	0.003	0.803
53	PF3D7_1126200	40S ribosomal protein S18, putative	12	0.002	0.802

54	PF3D7_1129000	spermidine synthase	47	0.015	0.899
55	PF3D7_1132200	T-complex protein 1 subunit alpha	20	1.068	0.955
56	PF3D7_1218500	Dynamin-like protein, putative	28	0.027	0.873
57	PF3D7_1218600	Arginine – tRNA ligase	7	0.717	0.874
58	PF3D7_1222100	Conserved plasmodium protein, unknown function	17	0.021	0.893
59	PF3D7_1229500	T-complex protein 1 subunit gamma	25	0.131	0.907
60	PF3D7_1238800	Acyl-CoA synthetase	61	0.008	0.887
61	PF3D7_1241700	Replication factor C subunit 4, putative	9	0.033	0.928
62	PF3D7_1242800	Rab specific GDP dissociation inhibitor	9	0.171	0.915
63	PF3D7_1252100	Rhoptry neck protein 3	161	0.089	0.897
64	PF3D7_1320000	Golgi protein 1	31	0.004	0.910
65	PF3D7_1320600	Ras-related protein Rab-11A	43	0.061	0.917
66	PF3D7_1323600	Conserved protein, unknown function	19	0.024	0.874
67	PF3D7_1324900	L-lactate dehydrogenase	75	4.157	0.939
68	PF3D7_1338300	Elongation factor 1-gamma, putative	37	0.003	0.896
69	PF3D7_1342600	Myosin A	39	0.005	0.892
70	PF3D7_1345700	Isocitrate dehydrogenase (NADP), mitochondrial	35	0.000	0.851
71	PF3D7_1347500	DNA/RNA-binding protein Alba 4	39	0.012	0.870
72	PF3D7_1354500	Adenylosuccinate synthetase	8	0.008	0.899
73	PF3D7_1360800	Falcilysin	110	0.149	0.928
74	PF3D7_1402300	26S proteasome regulatory subunit RPN6	15	0.001	0.924
75	PF3D7_1407800	Plasmepsin IV	40	2.106	0.948
76	PF3D7_1408000	Plasmepsin II	57	0.045	0.890
77	PF3D7_1409800	CUGBP Elav-like family member 2, putative	15	0.124	0.920
78	PF3D7_1439900	Triosephosphate isomerase	24	0.004	0.905
79	PF3D7_1442300	tRNA import protein tRIP	14	0.001	0.872
80	PF3D7_1451100	Elongation factor 2	50	0.151	0.916
81	PF3D7_1461900	Valine – tRNA ligase, putative	8	0.004	0.856
82	PF3D7_1462800	Glyceraldehyde-3-phosphate dehydrogenase	66	0.022	0.914
83	PF3D7_1468700	Eukaryotic initiation factor 4A	19	0.010	0.925

PSMs number of peptide spectrum matches, *MDT* Mininal Dose Threshold, minimal drug dose required to induce protein stabilization, R^2 the dose-response curve fitting quality, *MS-CETSA* mass spectrometry-coupled cell thermal shift analysis
